# From environmental occurrence to human exposure: multisystem toxicity, immune-relevant mechanisms, and gaps in health-risk assessment of liquid crystal monomers

**DOI:** 10.3389/fpubh.2026.1977975

**Published:** 2026-10-12

**Authors:** Hongchi Zhang, Jingle Qiu, Zhenyu Lu, Xiangbin Zheng, Xia Huo, Xijin Xu

**Affiliations:** 1 Laboratory of Environmental Medicine and Developmental Toxicology, Shantou University Medical College, Shantou, Guangdong, China; 2 Center for Reproductive Medicine, Clinical Research Center, Shantou Central Hospital, Shantou, Guangdong, China; 3 Laboratory of Environmental Medicine and Developmental Toxicology, Guangdong Key Laboratory of Environmental Pollution and Health, School of Environment and Climate, Jinan University, Guangzhou, Guangdong, China; 4 Department of Cell Biology and Genetics, Shantou University Medical College, Shantou, Guangdong, China

**Keywords:** health-risk assessment, human biomonitoring, human exposure, immune-relevant mechanisms, liquid crystal monomers, multisystem toxicity

## Abstract

Liquid crystal monomers (LCMs), functional constituents of liquid crystal displays, are emerging contaminants increasingly detected in environmental and human matrices, yet the implications of these exposures for human health remain uncertain. This review critically synthesizes current evidence on environmental occurrence and potential exposure pathways, human biomonitoring, toxicokinetics, multisystem toxicity, immune-relevant mechanisms, and health-risk assessment gaps. PubMed, Web of Science Core Collection, and Scopus were searched from database inception to 22 July 2026. Forty-one primary studies met the predefined eligibility criteria, while additional environmental-occurrence studies were used to contextualize sources, release processes, and potential exposure pathways. LCMs have been reported in dust, airborne particulate matter, soils, sediments, and landfill leachate, indicating several plausible exposure pathways, including dust ingestion, inhalation, dermal contact, and dietary intake. LCMs have been detected in urine, serum, human milk, maternal serum, and cord serum, indicating exposure in the general population and during sensitive life stages. Experimental studies report neurological, hepatic, renal, intestinal, respiratory, cutaneous, endocrine, reproductive, developmental, and metabolic effects. Oxidative stress, inflammatory signaling, nuclear receptor perturbation, metabolic dysregulation, and gut microbiota disruption provide biological plausibility for immune-related effects, although direct evidence of classical immunotoxicity remains limited. Major uncertainties include heterogeneous analyte coverage, insufficient human toxicokinetic data, limited chronic low-dose and mixture studies, and the absence of congener-specific reference values. Future research should prioritize harmonized biomonitoring, environmentally relevant mixture testing, prospective studies in susceptible populations, and integrated risk-assessment frameworks for this emerging class of contaminants.

## Graphical Abstract

**Figure:**
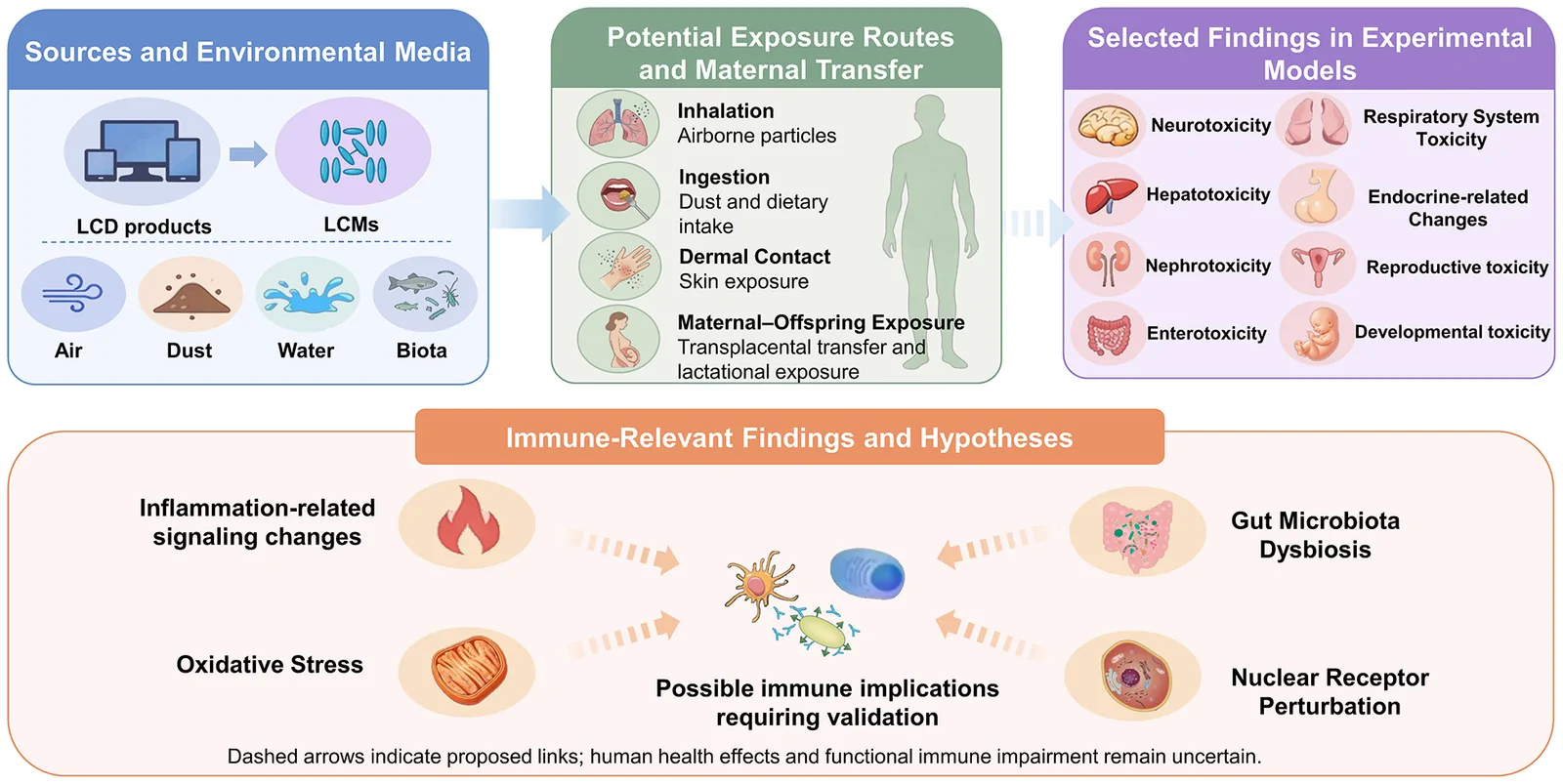

## 1 Introduction

Liquid crystal displays (LCDs) are widely used in modern consumer electronics, including smartphones, tablets, televisions, laptops, and wearable devices. Rapid product turnover has accelerated global generation of electronic waste (e-waste). According to The Global E-waste Monitor 2024, 62 million tons of e-waste were generated globally in 2022, of which only 22.3% was documented as formally collected and recycled in an environmentally sound manner (1). This rapid accumulation of e-waste has heightened concerns about chemical releases during LCD manufacturing and end-of-life disposal. Among these chemicals, liquid crystal monomers (LCMs)—the functional constituents of LCD panels—have attracted growing attention as an emerging class of contaminants with persistence, bioaccumulation potential, and toxicity (2–5).

LCMs comprise a structurally diverse family of compounds used as functional components of liquid crystal display panels (2, 6, 7). Their molecular structures generally contain rigid aromatic and/or cycloaliphatic cores bearing alkyl, alkoxy, cyano, and/or fluorine substituents, giving rise to numerous congeners with distinct structural features. Representative compounds discussed in this review include 3,4-difluoro-4′-(trans-4-ethylcyclohexyl)biphenyl (DFECB), 1-ethoxy-2,3-difluoro-4-(trans-4-propylcyclohexyl)benzene (EDPrB), 4-cyano-4′-ethoxybiphenyl (EBCN), 4-(4-ethylcyclohexyl)benzonitrile (EtCBN), 4′-(hexyloxy)-4-biphenylcarbonitrile (6OCB), and 4″-ethyl-2′,3,4,5-tetrafluoro-1,1′:4′,1″-terphenyl (2teFT). Together, these compounds illustrate several recurring structural features of LCMs, including fluorine and cyano substituents as well as biphenyl, terphenyl, and cycloaliphatic cores, as shown in Figure 1. Because many LCMs are physically incorporated rather than covalently bound within display materials, they may be released during manufacturing, product use, disposal, dismantling, recycling, and informal e-waste processing (8, 9). LCMs have been detected in indoor dust, ventilation and air-conditioning filter dust, airborne particulate matter, soils, sediments, and municipal landfill leachate (7, 10–16). These media may contribute to human exposure through dust ingestion, inhalation of particle-associated LCMs, dermal contact with contaminated dust or surfaces, and dietary intake (13, 16, 17). Together, these environmental sources, media, and potential pathways provide the source-to-exposure context needed to interpret the biomonitoring and toxicological evidence reviewed below.

**Figure 1:**
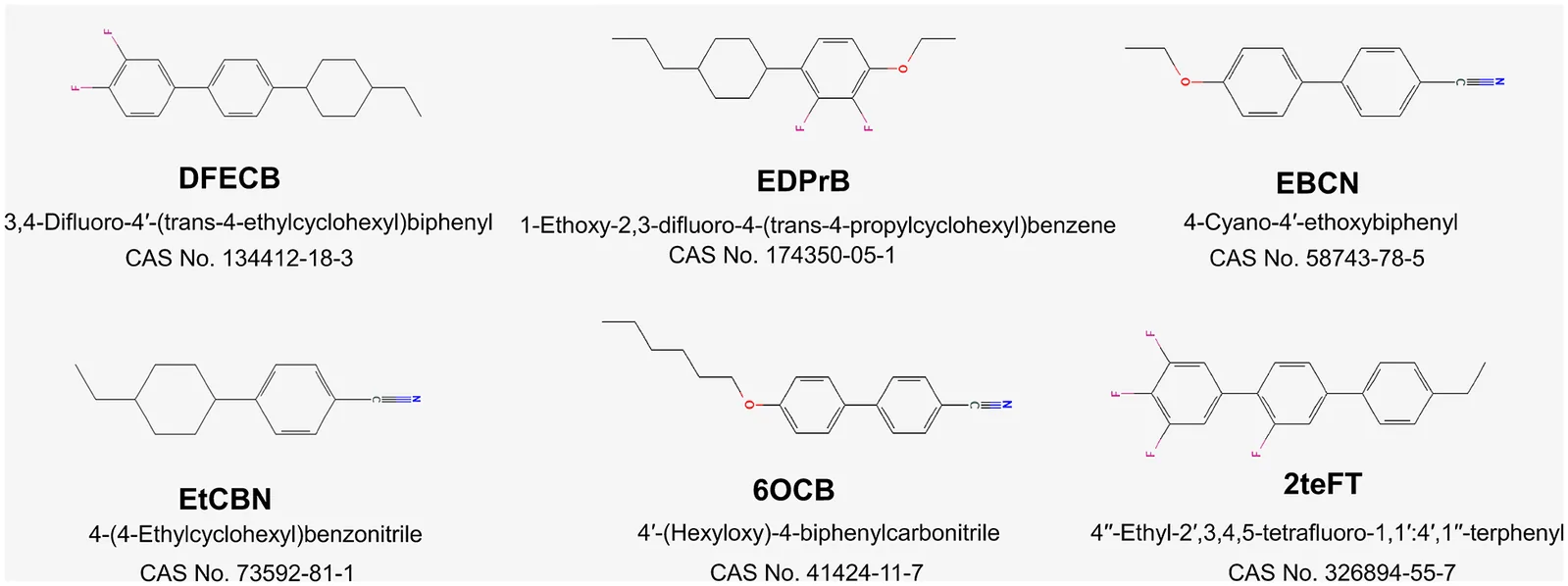
Representative chemical structures of selected liquid crystal monomers (LCMs). The compounds shown illustrate several recurring structural features of LCMs, including fluorine and cyano substituents as well as biphenyl, terphenyl, and cycloaliphatic cores. Additional compound-level information, including nomenclature, exposure conditions, and toxicological findings, is provided in Supplementary Table S2.

Human biomonitoring has detected LCMs in serum, urine, human milk, maternal serum, and cord serum, documenting internal exposure in occupational and general-population settings and during pregnancy and early life (18–25). However, these measurements do not by themselves identify the predominant exposure sources or routes, establish the duration of exposure, or demonstrate adverse health effects.

Evidence on absorption, distribution, metabolism, excretion, and toxicity (ADMET) provides a complementary basis for interpreting these measurements. Paired maternal and cord serum analyses support placental transfer of selected LCMs, while animal studies demonstrate compound-dependent tissue uptake and distribution, including accumulation in the brain and liver (22, 26, 27). Experimental studies also report oxidative stress, metabolic disturbance, endocrine disruption, and neurodevelopmental effects (28–30). Nevertheless, human absorption fractions, metabolic pathways, elimination kinetics, and relationships between external exposure and target-tissue dose remain insufficiently characterized (3, 4).

Despite these advances, potential effects on the immune system remain insufficiently characterized (3, 4, 31). Nevertheless, mechanistic observations, including oxidative stress, inflammatory signaling, endocrine disruption, metabolic reprogramming, and receptor interference, provide a biologically plausible rationale for immune-related effects (3, 4, 28, 29). While these findings do not establish immunotoxicity, they justify further investigation of possible immune-related effects.

An emphasis on immune-relevant mechanisms is supported by recurring findings across experimental studies of LCMs. These include oxidative stress and inflammatory signaling in renal and hepatic models (28, 32, 33), nuclear receptor perturbation and altered lipid metabolism (28, 34), and gut microbiota disruption and intestinal barrier injury (35–37). Together, these findings provide a basis for examining how processes relevant to immune regulation may connect toxic effects across organ systems. They do not, however, establish functional immune impairment. We therefore use immune-relevant mechanisms as a framework for integrating the available evidence, while distinguishing these observations from direct assessments of immune-cell function, immune-organ pathology, and host defense.

Previous reviews have summarized the environmental occurrence, release, fate, exposure pathways, and toxicity of LCMs (3–5). Building on this work, the present review integrates evidence on human biomonitoring, maternal–fetal and lactational exposure, toxicokinetics, and organ-specific toxicity, while distinguishing direct immune effects from mechanisms with potential relevance to immune function. We critically assess the strength and relevance of this evidence to human health and identify priorities for health-risk assessment.

Notably, environmental management and health-risk assessment of LCMs remain underdeveloped relative to the growing body of research on exposure and toxicity. More specifically, route-specific exposure estimates, congener-specific reference values, and standardized frameworks for health-risk assessment remain limited (3–5).

Therefore, this review critically synthesizes current evidence on environmental occurrence, potential exposure pathways, human exposure, biomonitoring, toxicokinetics, multisystem toxicity, immune-relevant mechanisms, and health-risk assessment gaps associated with LCMs. Rather than treating immunotoxicity as an established endpoint, we evaluate immune-relevant mechanisms as an integrative framework for interpreting convergent findings across organ systems. By distinguishing direct toxicological evidence from mechanistic plausibility, this review aims to identify major hazards, highlight potentially susceptible populations and priority exposure scenarios, and define priorities for future environmental monitoring, route-specific exposure assessment, biomonitoring, mixture testing, mechanistic research, and health-risk assessment.

## 2 Literature search and evidence synthesis

PubMed, Web of Science Core Collection, and Scopus were searched from database inception through 22 July 2026 for English-language records relating to LCM exposure, biomonitoring, toxicokinetics, toxicity, and immune-relevant mechanisms. Terms identifying LCMs were combined with a second concept block comprising either environmental occurrence and exposure terms or toxicological and mechanistic terms. Synonyms within each concept were combined using OR, and the LCM and exposure/toxicity concept blocks were linked using AND. Web of Science Core Collection and Scopus were restricted to English-language articles, whereas the English-language restriction was incorporated directly into the PubMed search. The complete database-specific search expressions, search fields, filters, search dates, and record counts are provided in Supplementary Table S1.

Original peer-reviewed studies were eligible for the primary evidence synthesis if they reported LCM concentrations in human biological samples, quantitative estimates of human exposure, toxicokinetics or tissue distribution in mammalian or zebrafish models, or toxicological effects in mammalian, zebrafish, mammalian-cell, or organoid models. Studies examining direct immune endpoints or biological processes relevant to immune regulation were also eligible. Computational studies were included only when their predictions were supported by experimental validation conducted within the same study. Non-original and non-peer-reviewed publications were excluded, as were studies concerned exclusively with materials chemistry, physicochemical characterization, display applications, treatment or removal processes, or non-monomeric liquid-crystal materials. Environmental-occurrence studies without quantitative estimates of human exposure were used as contextual evidence in Section 3.1 but were not included in the primary-study count. When two or more publications reported substantially overlapping datasets, the most complete report was retained.

All records were imported into EndNote 20. Duplicate records were identified manually by comparing titles, authors, publication details, and digital object identifiers. Two reviewers (H.Z. and Z.L.) independently screened the titles and abstracts and subsequently assessed the full texts of potentially eligible reports. Disagreements were resolved through discussion; cases in which consensus could not be reached were adjudicated by a third reviewer (J.Q.). The principal reason for exclusion was recorded for each report excluded at the full-text assessment stage. Extracted information included publication year, study design, study population or experimental model, compound identity or mixture composition, exposure matrix or route, dose and exposure duration, toxicokinetic and toxicological findings, proposed mechanisms, and major methodological limitations. Each eligible publication was counted once, even when it contributed evidence to more than one section of the review.

This review was undertaken as a critical narrative synthesis rather than a systematic review. Its purpose was to integrate heterogeneous evidence spanning environmental occurrence, human exposure, toxicokinetics, organ-specific toxicity, and immune-relevant mechanisms and to evaluate the strengths and limitations of the connections between these evidence streams, rather than to answer a single, narrowly framed exposure–outcome question or generate a pooled quantitative estimate. In this context, “critical” refers to the explicit appraisal of study design, exposure conditions, model relevance, consistency across studies, replication, and the extent of experimental validation supporting the reported findings. The review protocol was not prospectively registered, and no formal study-level risk-of-bias tool was applied. Evidence was therefore synthesized narratively and appraised qualitatively. Biomonitoring measurements were interpreted as evidence of internal exposure without being taken to demonstrate adverse health effects, whereas experimental findings were evaluated primarily for their contribution to hazard identification and mechanistic understanding. Direct immune endpoints were distinguished from inflammatory, endocrine, metabolic, barrier-related, and microbiota-associated processes with potential relevance to immune regulation. No formal numerical scoring system was used.

The database searches identified 661 records, of which 595 remained after application of the specified language and article-type restrictions: 151 from PubMed, 217 from Web of Science Core Collection, and 227 from Scopus. After 323 duplicate records had been removed, 272 unique records underwent title and abstract screening, of which 175 were excluded. The full texts of all 97 remaining reports were retrieved and assessed for eligibility. Fifty-six reports were excluded at the full-text stage, leaving 41 unique primary studies for inclusion in the qualitative synthesis. Figure 2 summarizes the study-selection process and the principal reasons for full-text exclusion.

**Figure 2:**
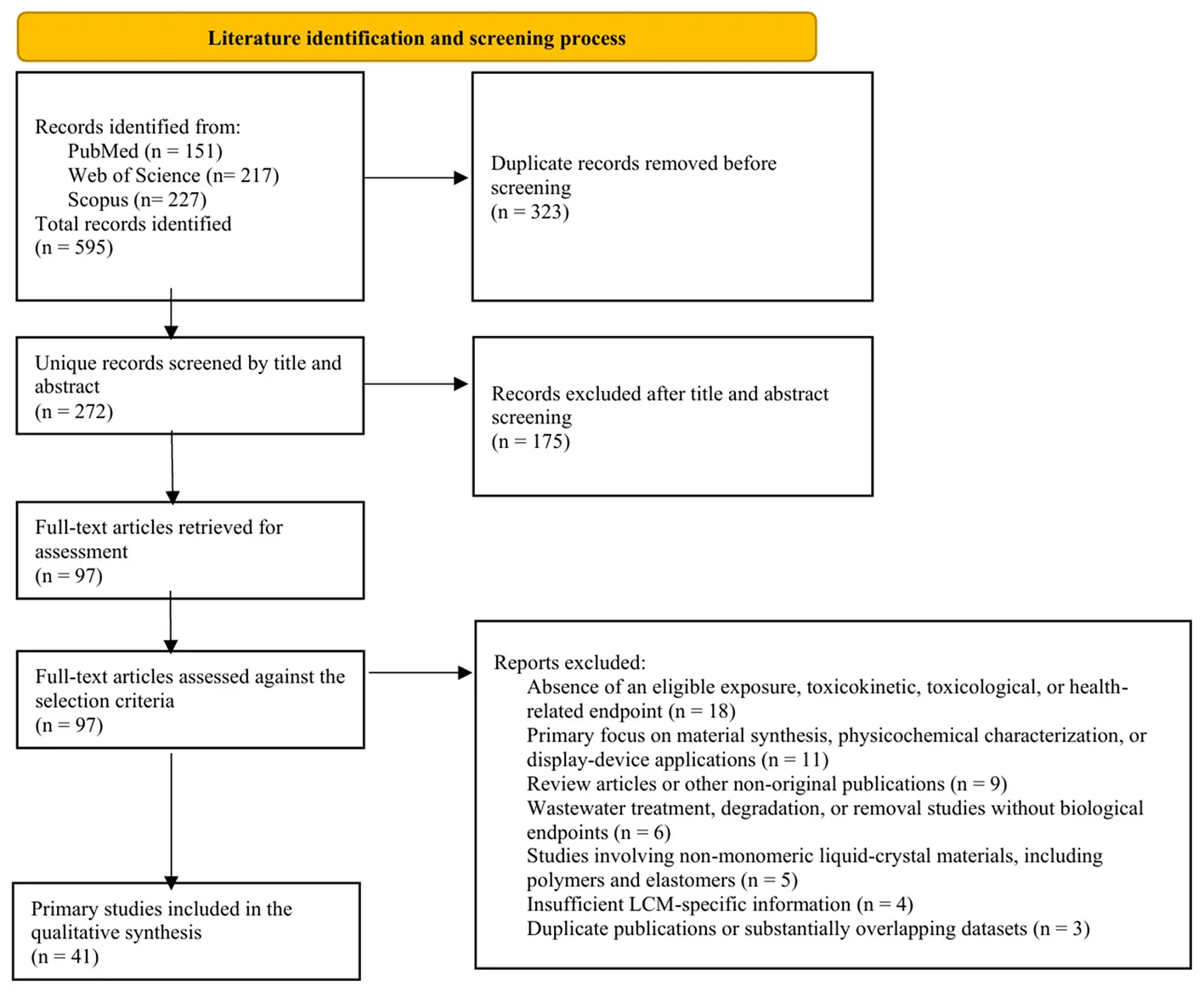
Literature identification and selection flow diagram.

## 3 Environmental occurrence, human exposure, and toxicokinetic evidence

### 3.1 Environmental sources, occurrence, and potential exposure pathways

LCD manufacturing, product use, and end-of-life dismantling and recycling can release LCMs into the environment (2, 6, 8, 9). LCMs have been detected in dust, airborne particulate matter, soils, sediments, and landfill leachate in residential, commercial, and industrial settings (10–14, 16, 38–42). Their concentrations and congener profiles vary across these settings, providing a basis for examining potential sources and pathways of human exposure.

Quantitative dust measurements demonstrate differences in both contamination levels and congener profiles across microenvironments. In a nationwide Chinese study targeting 60 LCMs, median total concentrations were 41.6 ng/g in residential indoor dust, 94.7 ng/g in residential outdoor dust, 106 ng/g in cybercafé dust, and 171 ng/g in phone-repair-store dust (16). EtCBN and PBIPHCN were the predominant congeners in residential indoor dust, with median concentrations of 20.6 and 13.0 ng/g, respectively. By contrast, ETFMBC predominated in cybercafé dust, whereas BDFTFMTFT predominated in phone-repair-store dust (16). In an e-waste recycling industrial park in Central China, median total concentrations of the 55 target LCMs were 6950 ng/g in outdoor dust and 67,400 ng/g in indoor dust from the main dismantling workshop (43). These findings highlight the importance of distinguishing residential, commercial, and occupational environments when interpreting LCM occurrence.

Compound profiles also vary between environmental phases. In educational and workplace indoor air, Lin et al. (13) identified 4-propyl-4′-vinylbicyclohexyl (3VbcH) and 4,4′-bis(4-propylcyclohexyl) biphenyl (b3CHB) as the predominant LCMs in the gaseous and particulate phases, with median concentrations of 101 and 1460 pg/m^3^, respectively. These findings indicate that environmental occurrence should be characterized by both congener composition and phase-specific concentrations.

LCMs in these media may contribute to several plausible pathways of human exposure. Dust ingestion and dermal contact may be particularly relevant in homes, workplaces, campuses, and e-waste recycling settings, whereas particle-associated LCMs may contribute to inhalation exposure. Quantitative studies have estimated LCM exposure through dust ingestion, dermal contact, and inhalation (13, 16, 43). For airborne LCMs in educational and workplace environments, probabilistic exposure modeling indicated that inhalation exposure generally exceeded dermal absorption by approximately one to two orders of magnitude, although dermal absorption was comparable to or greater than inhalation for certain compounds (13). This comparison applies to the airborne exposure scenarios evaluated and does not establish the dominant pathway across all environmental media or populations. Measurements of indoor and outdoor PM10 provide additional evidence that LCMs occur in inhalable particle-associated fractions (44). Indoor–outdoor contrasts in dust contamination vary among studies and sampling settings, and concentration differences should be distinguished from differences in modeled intake, which also depend on exposure frequency and behavioral assumptions (16, 45).

Experimental investigation of emissions from electronic devices further supports a source-to-exposure pathway linking indoor display-containing products with LCM accumulation in indoor air and dust (46). Maternal–fetal and lactational transfer should be distinguished from these primary environmental exposure routes because they occur after maternal internal exposure.

Dust-based exposure estimates also indicate differences between age groups and exposure settings. For residential indoor dust in the nationwide study, estimated daily intakes under the typical (P50) exposure scenario were 9.57 × 10^−3^ and 1.89 × 10^−2^ ng/kg body weight/day through ingestion and dermal contact, respectively, for adults; corresponding estimates for children were 1.23 × 10^−1^ and 1.02 × 10^−1^ ng/kg body weight/day (16). In the e-waste recycling study, median estimated daily intakes for dismantling workers were 48.3 ng/kg body weight/day through dust ingestion and 16.5 ng/kg body weight/day through dermal absorption, compared with 0.19 and 0.15 ng/kg body weight/day, respectively, for reference residents (43). These model-based estimates characterize potential exposure under the specified assumptions rather than measured internal doses or demonstrated adverse health effects.

Environmental detection, however, does not by itself establish the magnitude of human exposure or the contribution of any single pathway. Differences in target analytes, sampling media, analytical methods, and exposure models limit direct comparisons across studies. In particular, the dust-based studies used a dermal absorption factor of 0.13 adapted from other chemicals with similar hydrophobicity because LCM-specific values were unavailable (16, 43). Furthermore, inhalation estimates derived from settled-dust concentrations and a particle emission factor should not be directly equated with estimates based on measured airborne LCMs (13, 16). Although individual studies provide scenario-specific estimates, the available evidence does not establish the relative contributions of dust ingestion, inhalation, dermal contact, and dietary intake to aggregate LCM exposure across populations. Available measurements remain predominantly concentrated in China, although recent evidence from Pakistan has begun to expand the geographical scope of LCM exposure assessment. Accordingly, environmental occurrence data should be interpreted as source-to-exposure evidence that complements, rather than substitutes for, human biomonitoring.

### 3.2 Human biomonitoring evidence

Human biomonitoring studies consistently document internal exposure to LCMs across occupational and general-population settings, including pregnancy and early life (18–25, 47, 48). Their main contribution is to establish that environmental exposure results in measurable concentrations in human biological samples. However, the magnitude and composition of this exposure vary across populations and matrices, and the available measurements do not establish the predominant exposure routes or the likelihood of adverse health effects. Table 1 summarizes study characteristics and concentration data; the synthesis below focuses on patterns shared across studies and the limitations of comparisons between them.

**Table 1:** Summary of human biomonitoring evidence for LCM exposure.

Human Samples	Location	Date	Population	Sample size	Gender (Male/Female)	Age (Years)	Detection	Reported concentration	Major or frequently detected LCMs^a^	References
Urine	Guiyu, Guangdong, China (E-waste Area)	NR	Children (Exposed)	96	62/34	2–7	30 targeted^b^	0.63–29.11 (median: 8.76)	PrEPCC; EBCN; BuCBN (major contributors)	(47)
	Haojiang, Guangdong, China		Children (Control)	98	45/53	2–7	30 targeted^b^	0.05–22.64 (median: 5.36)	EBCN; BuCBN; PrEPCC (major contributors)	
	Beijing, China (General Population)	November 2020 to February 2021	Mothers	95	0/95	27–37	34 LCMs	0.47–10.40 (median: 1.28)	BDPrB; EFPEB (prominent congeners)	(21)
			Infants	95	48/47	0–12 months	35 LCMs	0.26–16.20 (median: 0.60)	BDPrB (DF: 100%); EFPEB (prominent congener)	
	Dingxi, Gansu, China	July 2022	General Population	169	NR	NR	14 detected; 15 targeted	1.97–208.20 (median: 28.58)	BEEB; EBCN (major contributors)	(48)
Serum	Beijing, China (General Population)	August 2017 to November 2018	General Population	314	141/173	19–74	38 LCMs	ND−31.38 ng/mL (median: 0.92 ng/mL)	BDPrB (highest DF: 85.7%)	(19)
	Guangdong, China (E-waste Area)	2015	E-waste workers (Exposed)	85	NR	≥60	29 LCMs	7.78–276 (median: 35.2)	BCBP; EBCN; PrTFMPBC; FPrCB (major contributors)	(18)
			Urban residents (Control)	16	10/6	≥60	10 LCMs	3.16–28.50 (median: 9.59)	EDFPCB; PBIPHCN (major contributors)	(18)
	Qingyuan, Guangdong, China (E-waste Area)	2016 to 2017	Mothers	93	0/93	18–37	14 LCMs	4.85–231 (median: 13.90)	BDFPrCB (highest median conc.)	(22)
			Infants (Cord Serum)	93	33/23;37 missing	38–42 gestational weeks	14 LCMs	3.91–175 (median: 18.10)	BDFPrCB (highest median conc.)	(22)
	Dingxi, Gansu, China	July 2022	General Population	163	NR	NR	15 LCMs	15.22–380.19 (median: 126.55)	BEEB (highest mean conc.)	(48)
	Jiangsu Province, Eastern China	2019	General Population	112	NR	9–80	36 LCMs (42 targeted)	0.041–127 (median: 10.7)	5OCB; 8OCB; 2bcHdFB; 5CT (major contributors)	(23)
Human milk	Beijing, China	November 2020 to February 2021	Lactating mothers	93	0/93	22–43	30 LCMs	11.97–2.82 × 10^4^ ng/g lw (median: 133.40 ng/g lw)	BDPrB (predominant contributor)	(20)
	Hunan Province, China	2011 to 2012	Lactating mothers	80	0/80	18–36	58 LCMs (78 targeted)	5.00–358 ng/g lw (median: 27.1 ng/g lw)	EDPeB (predominant contributor)	(25)
	China, 24 provinces	2017 to 2019	Primiparous lactating mothers	100 pooled samples (from 3467 donors)	0/3467 donors	NR	17 detected; 38 targeted^c^	0.129–23.4 (median: 2.49)	MOPB (highest median conc.)	(24)

LCMs, liquid crystal monomers; lw, lipid weight; DF, detection frequency; ND, not detected; NR, not reported; conc., concentration. Units: ng/mL unless specified; concentration bases are not directly comparable. ^a^Congeners are listed by concentration contribution, mean or median concentration, or detection frequency, as indicated; frequent detection does not imply predominance by concentration. ^b^30 targets; individual DFs reported (47). ^c^Sum includes 15 congeners with DF >10% (24). Dingxi data: original Tables 2, 3 (48). Milk pools combine same-site donors. Biomonitoring indicates exposure, not established harm or causation.

Within-study comparisons suggest that e-waste-related activities may contribute to elevated internal exposure. Median total serum LCM concentrations were higher in dismantling workers than in reference residents in Guangdong (35.2 versus 9.59 ng/mL) (18). A similar pattern was observed in children: median total urinary concentrations were 8.76 ng/mL in Guiyu, an e-waste recycling area, and 5.36 ng/mL in Haojiang, a reference area without e-waste recycling activities (47). These comparisons are consistent with greater exposure in e-waste settings, but the observational designs do not isolate the contribution of individual sources or exposure routes. Detection in the reference groups also indicates that exposure extends beyond populations directly associated with e-waste recycling.

Comparisons across general-population studies are less straightforward. Reported median total serum concentrations ranged from 0.92 ng/mL in Beijing to 10.7 ng/mL in Jiangsu and 126.55 ng/mL in Dingxi (19, 23, 48). These differences cannot be interpreted as a geographic exposure gradient because the studies differed in participant characteristics, sampling periods, analytical methods, and target analytes. The Beijing study examined fluorinated liquid crystal monomers (FLCMs), whereas the Jiangsu and Dingxi studies also included non-fluorinated compounds, which contributed substantially to the measured totals (19, 23, 48). Thus, “total LCM concentration” is a study-specific sum rather than a uniform measure across the literature. Congener profiles likewise provide complementary information: BEEB predominated in Dingxi serum, whereas several other compounds, including 5OCB and 8OCB, contributed prominently in Jiangsu (23, 48). Differences in analytical coverage must therefore be considered before attributing these profiles to regional sources or biological processes.

Evidence from paired samples is particularly informative for exposure during sensitive life stages. In 93 mother–infant pairs from Qingyuan, median total LCM concentrations were 13.90 ng/mL in maternal serum and 18.10 ng/mL in cord serum, supporting transplacental transfer of selected congeners (22). These findings establish fetal exposure but do not, by themselves, demonstrate developmental harm. Postnatal exposure is also documented by measurements in maternal and infant urine and in human milk (20, 21, 24, 25). Interpretation of urine data depends on the reporting metric: in the Beijing mother–infant study, maternal concentrations were higher on a volume basis, whereas infant concentrations were higher after creatinine adjustment (21). This reversal shows why comparisons between age groups should not rely on a single concentration metric.

Human-milk studies collectively document potential lactational exposure, but they do not identify a consistently dominant congener across populations. BDPrB predominated in the Beijing study, EDPeB in Hunan, and MOPB in the national survey (20, 24, 25). These differences may partly reflect analytical coverage; notably, MOPB was not included in the Hunan target panel (24, 25). Study design further limits comparison: the Beijing and Hunan studies analyzed individual samples and reported lipid-normalized concentrations, whereas the national survey analyzed pooled samples and reported concentrations per unit volume. Pooling also limits assessment of variation among individual donors. Moreover, milk concentrations alone cannot quantify maternal-to-infant transfer efficiency without paired maternal measurements and information on milk intake.

Paired environmental and biological measurements offer some evidence linking external contamination with internal exposure, although congener profiles need not match. In the Beijing dust–milk study, BDPrB contributed much more strongly to the milk profile than to the dust profile, and concentrations of seven FLCMs were positively correlated between paired samples (20). These findings are consistent with a contribution from household contamination but do not establish dust as the dominant exposure source or identify the route of uptake. Differences between environmental and biological profiles may also reflect congener-specific absorption, distribution, metabolism, and excretion.

Overall, the evidence supports internal exposure across the populations studied, with indications of elevated exposure in e-waste settings and direct evidence of exposure during fetal and early postnatal life. The principal limitation is the lack of comparability across studies, rather than a lack of detection. Harmonized analyte panels, clearly defined concentration sums, consistent reporting of volume-based and normalized measurements, and repeated paired sampling are needed to distinguish population differences from methodological variation and to better connect external exposure with internal concentrations.

### 3.3 Emerging human health-related evidence

Human evidence linking LCM exposure to health-related outcomes remains preliminary. A cross-sectional study of children from an e-waste recycling area and a reference area reported associations between specific LCM classes and anthropometric measures (47). Among children in the high-exposure group identified by clustering urinary LCM profiles, higher urinary concentrations of fluorinated biphenyls and analogs (FBA) were associated with greater head and chest circumference after full covariate adjustment. Higher concentrations of cyanobiphenyls and analogs (CBA) were associated with greater head circumference, but not chest circumference. In stratified analyses, positive associations with head circumference were observed for both classes in girls and for CBA in children younger than 5 years. In a Beijing study of the general population, serum concentrations of EFPEB, EDPrB, EDFPBB, and PDTFMTFT were positively correlated with blood glucose (*r* = 0.126–0.275, *p* < 0.05) (19). These studies extend human biomonitoring beyond exposure characterization, but their findings should be considered hypothesis-generating because of cross-sectional designs, single-time-point measurements, heterogeneous analyte panels, multiple comparisons, and potential confounding by co-exposures and lifestyle factors. Prospective studies with repeated exposure measurements and prespecified clinical or developmental outcomes are therefore required.

### 3.4 Animal toxicokinetic and tissue-distribution evidence

Animal studies show that LCMs distribute to multiple tissues, although their accumulation and elimination differ among compounds. Following oral administration in mice, 3dFB, 2OdF3B, 2teFT, and 6OCB were detected in the liver, adipose tissue, and brain, among other tissues (27). Of the compounds tested, 2teFT showed relatively high accumulation and slow elimination, while 6OCB accumulated more than 3dFB despite their similar hydrophobicity (27). In adult zebrafish exposed to a mixture of 39 LCMs, total LCM concentrations were highest in the intestine, followed by the brain, gills, liver, and muscle (26). This order reflects summed concentrations across the mixture, not the distribution of each individual compound. The studies therefore identify common sites of distribution but do not establish that all LCMs accumulate predominantly in the same organs. Differences in species, exposure conditions, and sampling times also limit comparisons between studies. Tissue accumulation alone should not be interpreted as evidence of toxicity.

## 4 Major toxic effects of liquid crystal monomers

Studies indicate that selected LCMs can induce oxidative stress, metabolic and endocrine disruption, developmental toxicity, and neurotoxicity in experimental models (3). Tissue distribution and trans-barrier transport provide complementary toxicokinetic evidence relevant to interpreting these effects (22, 26, 27). As summarized in Figure 3, experimentally observed effects and proposed immune-relevant mechanisms span the nervous system, liver, kidneys, intestine, respiratory tract, skin, endocrine and reproductive systems, as well as metabolic processes across multiple tissues. These findings differ in evidentiary role and should be interpreted within the experimental models and exposure conditions investigated. Reported effects vary among compounds and experimental models, but the lack of standardized comparisons limits conclusions about differences in potency, target-organ selectivity, or mechanisms of action across structural classes (3). Expanded compound identities, experimental models, exposure concentrations and durations, exposure-context information, and detailed toxicological endpoints are provided in Supplementary Table S2. Accordingly, this review synthesizes the evidence by biological system and tissue to clarify the major endpoints and mechanisms. Because metabolic disturbance appears repeatedly across hepatic, renal, endocrine, developmental, and computational toxicology studies, metabolic toxicity is discussed here as a cross-organ toxicological theme in addition to the system- and tissue-specific endpoints summarized below (28, 32, 34, 49, 50).

**Figure 3:**
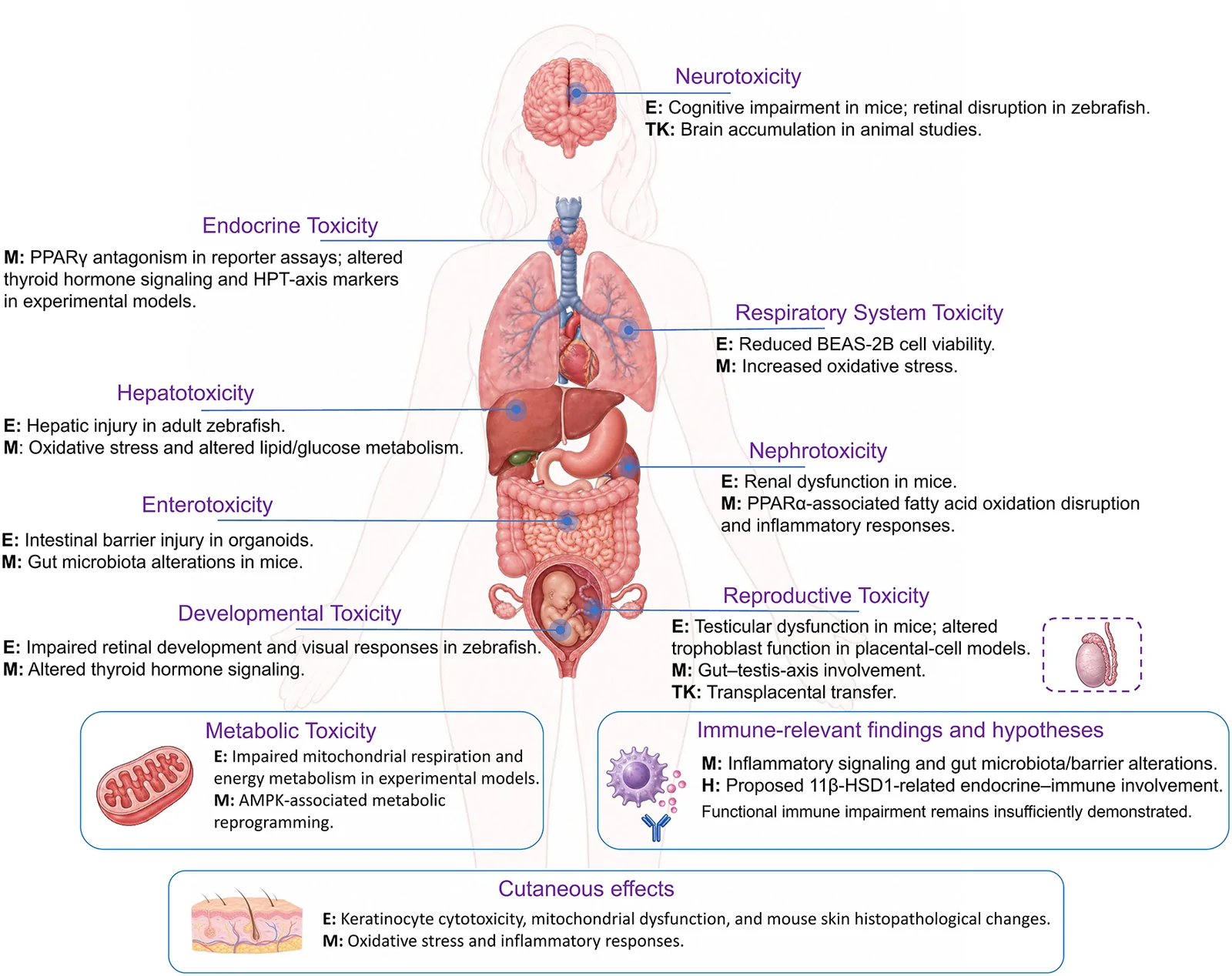
Experimentally observed effects, mechanistic findings, and proposed immune-relevant links of liquid crystal monomers across biological systems. Findings apply to the compounds, experimental models, and exposure conditions investigated. E, experimentally observed effects; M, mechanistic findings or associations; H, hypothesis-generating evidence; TK, toxicokinetic observations. These labels indicate evidentiary roles rather than formal evidence-quality scores. Tissue accumulation and transplacental transfer are toxicokinetic observations and do not, by themselves, establish organ toxicity. The human-body illustration serves as an anatomical guide and does not indicate demonstrated adverse effects in humans. Proposed immune-relevant links require further validation, and functional immune impairment remains insufficiently demonstrated. Abbreviations: LCMs, liquid crystal monomers; PPARγ, peroxisome proliferator-activated receptor gamma; PPARα, peroxisome proliferator-activated receptor alpha; HPT axis, hypothalamic–pituitary–thyroid axis; AMPK, AMP-activated protein kinase; BEAS-2B, human bronchial epithelial cell line; 11β-HSD1, 11β-hydroxysteroid dehydrogenase type 1.

### 4.1 Neurotoxicity

The nervous system is one of the major targets of concern in current toxicological studies of LCMs. Experimental studies in animal models indicate that some LCMs can reach brain tissue and may induce developmental visual impairment, neuroinflammation, and cognitive dysfunction (26, 27, 29, 37).

Regarding developmental neurotoxicity, He et al. reported that exposure to six FLCMs, including 2O3cHdFP, tFPO-CF2-dF3B, 2O3cHdFB, 2OdFP3bcH, TPrCB, and BDPrB, disrupted retinal structure in zebrafish larvae, reduced cell densities in the ganglion cell layer, inner nuclear layer, and photoreceptor layer, and significantly inhibited visual motor responses under red and green light conditions (29). Further analyses indicated that these effects were closely associated with disrupted thyroid hormone signaling, suggesting that FLCMs may induce developmental neurotoxicity through an endocrine-disruption-mediated neurodevelopmental pathway (29).

With respect to neurotoxicity in adults, Duan et al. (37) established an 8-week mouse exposure model using 4-(4-ethylcyclohexyl) benzonitrile (EtCBN) as a representative compound and showed that exposure at 20 μg/kg, described by the authors as environmentally relevant, impaired cognitive function, while 200 μg/kg represented a higher experimental dose. *In vitro* experiments further demonstrated direct toxicity of EtCBN toward brain microvascular endothelial cells, microglia, and hippocampal neuron-like cells (37). By integrating hippocampal transcriptomics, plasma metabolomics, gut microbiota 16S rRNA sequencing, and microbiota-derived metabolite profiling, Duan et al. (37) further showed that EtCBN disrupted gut microbial composition, remodeled metabolic profiles, and activated the NOD-like receptor signaling pathway, thereby triggering neuroinflammation and impairing hippocampal synaptic plasticity. These findings converge to indicate that the neurotoxicity of LCMs may involve not only structural and functional impairment, but also immune-inflammatory responses, gut–brain axis dysregulation, and synaptic dysfunction.

Taken together, the available studies indicate that LCM-associated neurotoxicity may involve distinct manifestations across developmental and adult life stages. In early life, reported effects include impaired retinal development and altered sensory function, whereas adult exposure has been associated with neuroinflammation, gut–brain axis perturbation, and cognitive deficits (29, 37). Although endocrine disruption, inflammatory signaling, and brain tissue distribution emerge as recurring features across these studies, the extent to which these pathways represent shared mechanisms across life stages remains uncertain. Moreover, most evidence is derived from zebrafish and experimental mouse models, with limited direct evidence from human populations. These findings therefore provide evidence of neurotoxic potential and mechanistic plausibility rather than a definitive characterization of neurotoxicity in humans.

Experimental tissue-distribution studies further support the biological plausibility of nervous-system exposure, with LCM accumulation in brain tissue reported in animal models, as summarized in Section 3.4 (26, 27).

### 4.2 Hepatotoxicity

The liver is considered one of the primary target organs for which evidence of toxicity from LCMs is relatively well established. Using EDPrB (1-ethoxy-2,3-difluoro-4-(trans-4-propylcyclohexyl) benzene), a representative FLCM, as the test compound, Li et al. (32) conducted a 21-day exposure experiment in adult zebrafish using concentrations described by the authors as environmentally relevant and found that EDPrB induced abnormal liver morphology, accompanied by elevated transaminase activity and reduced antioxidant capacity. In addition, the exposed group also exhibited disturbances in lipid and glucose metabolism, together with activation of endoplasmic reticulum stress- and apoptosis-related pathways (32). The available evidence suggests that LCM-induced hepatotoxicity is not driven by a single mechanism, but rather reflects a combined response involving oxidative damage, metabolic dysfunction, endoplasmic reticulum stress, and cell death processes (32). Animal toxicokinetic studies also indicate preferential partitioning of several LCMs into the liver and adipose tissue, as summarized in Section 3.4 (27).

The evidence for hepatotoxicity is relatively consistent across experimental endpoints, particularly for oxidative stress, metabolic disturbance, and endoplasmic reticulum stress in exposed zebrafish. However, the available evidence remains dominated by experimental studies in non-mammalian models, and the extent to which the observed responses predict chronic hepatic effects in humans is uncertain. Differences in exposure duration, toxicokinetics, and dose comparability should therefore be considered when extrapolating these findings to human health.

### 4.3 Nephrotoxicity

Compared with neurotoxicity and hepatotoxicity, evidence regarding the renal toxicity of LCMs remains relatively limited but now includes both *in vivo* and *in vitro* mechanistic studies (28, 33).

Direct *in vivo* and mechanistic evidence was provided by Peng et al., who evaluated the renal toxicity of DFECB using integrated *in vivo, in vitro*, and *in silico* approaches. Female C57BL/6 mice orally exposed to DFECB at 10 mg/kg bw/day for 30 days exhibited increased serum blood urea nitrogen and creatinine levels, renal interstitial inflammation, glomerular morphological alterations, and disturbed renal metabolism. Metabolomic and molecular analyses further indicated inhibition of fatty acid β-oxidation and downregulation of peroxisome proliferator-activated receptor alpha (PPARα). Consistently, DFECB exposure in HK-2 cells induced oxidative stress, inflammatory responses, ATP depletion, a reduced NAD^+^/NADH ratio, and downregulation of fatty acid oxidation-related genes. Partial restoration of CPT1, CPT2, and ACSL1 expression by the PPARα agonist GW7647 supported disruption of PPARα-mediated fatty acid oxidation as a central mechanism of DFECB-induced renal dysfunction (33).

Complementary *in vitro* evidence was provided by Zhao et al. (28), who used HK-2 cells to show that selected LCMs may act as antagonists of peroxisome proliferator-activated receptor gamma (PPARγ) and induce marked transcriptomic and metabolomic disturbances in renal tubular epithelial cells. Among these alterations, dysregulation of fatty acid β-oxidation was particularly prominent, together with perturbation of multiple signaling pathways related to stress responses, inflammatory regulation, and energy metabolism, including interleukin-17 (IL-17), tumor necrosis factor (TNF), nuclear factor kappa B (NF-κB), and AMP-activated protein kinase (AMPK) signaling (28).

Together, these findings suggest that the renal effects of LCMs may extend beyond nonspecific cytotoxicity and involve coordinated disturbances in fatty acid metabolism, energy homeostasis, oxidative stress, and inflammatory signaling (28, 33). The combination of *in vivo* findings and mechanistically informative cell-based experiments strengthens the biological plausibility of these pathways. Nevertheless, human epidemiological evidence remains sparse, and uncertainty persists regarding chronic low-dose exposure, sex-specific susceptibility, congener-specific dose–response relationships, and the relevance of the experimental exposure scenarios to human exposure.

### 4.4 Enterotoxicity

Direct LCM-specific evidence is currently derived from mouse exposure studies, gut microbiota analyses, and intestinal organoid models. Among these, Wu et al. reported in a mouse study that long-term exposure to the representative liquid crystal monomer 4-cyano-4′-ethoxybiphenyl (EBCN) at concentrations described by the authors as environmentally relevant significantly reduced gut microbial diversity and decreased the production of the anti-inflammatory metabolite L-carnosine. This reduction was accompanied by elevated serum inflammatory cytokines, suggesting that gut microbial dysbiosis may contribute to systemic inflammatory imbalance and distal-organ effects (36). The reproductive manifestation reported in the same study is discussed in Section 4.9.

In addition, studies based on intestinal organoids and the adverse outcome pathway (AOP) framework further demonstrated that LCM mixtures can suppress organoid expansion and induce abnormal crypt budding, while machine-learning models identified EBCN as a potential contributor to the observed enterotoxic effects (35). Importantly, the predictive findings were followed by targeted exposure experiments showing that EBCN reduced cell viability, increased FITC-dextran permeability, decreased ZO1 and claudin expression, impaired fatty-acid uptake, and disrupted intestinal stem-cell homeostasis (35). Thus, the computational analysis served primarily as a tool for compound prioritization, whereas the subsequent experimental findings provided direct biological support for the predicted enterotoxic potential. Nevertheless, organoid and *in vitro* models cannot fully reproduce systemic toxicokinetics, immune-cell interactions, or whole-organism endocrine and metabolic regulation, and the implications of these findings for human gastrointestinal toxicity remain to be established.

Beyond the direct evidence described above, another study found that EtCBN exposure caused abnormal gut microbiota composition, significantly reduced the abundance of Muribaculum, and remodeled the plasma metabolomic profile, thereby activating the NOD-like receptor signaling pathway and ultimately inducing neuroinflammation and cognitive impairment (37). Although the primary outcome of that study was neurotoxicity, its findings indirectly support the bridging role of gut microbial dysbiosis in the systemic toxicity of LCMs.

### 4.5 Respiratory system toxicity

Current evidence regarding the respiratory toxicity of LCMs is mainly derived from airway epithelial cell models. Jin et al. (10) detected 31 LCMs in ventilation and air-conditioning filter (VACF) dust, with total concentrations ranging from 43.7 to 448 ng/g, and further selected 14 relatively frequently detected compounds (detection frequency >20%) for *in vitro* toxicity assessment using BEAS-2B cells. In that study, the BEAS-2B exposure concentrations were set at 0.34–34 μg/mL for individual LCMs and 0.1–10 units for the reconstructed mixture. These concentrations were derived from an estimated yearly intake model based on measured VACF dust concentrations and adult dust-ingestion parameters, with the mean yearly intake of the 14 LCMs corresponding to an exposure dose of 3.4 μg/mL for each compound (10). Therefore, the assay provides exposure-informed *in vitro* screening evidence for airway epithelial toxicity, although the tested concentrations should not be interpreted as directly equivalent to environmental dust concentrations because the latter are reported on a dust-mass basis. The results showed that these LCMs, whether tested individually or as mixtures, induced significant oxidative stress, and several non-fluorinated LCMs caused a more pronounced decrease in cell viability (10).

Because BEAS-2B cells are derived from human bronchial epithelium, these findings provide biologically relevant evidence that LCMs can induce oxidative and cytotoxic responses in airway epithelial cells (10). However, the *in vitro* exposure conditions cannot be directly equated with human inhalation exposure, and the contribution of these responses to respiratory disease in exposed populations remains uncertain.

### 4.6 Cutaneous toxicity

Recent evidence suggests that cutaneous tissue may also be affected by LCM exposure. Dongye et al. evaluated a five-LCM mixture using HaCaT human keratinocytes and a repeated topical-exposure model in male BALB/c mice. In HaCaT cells, exposure produced time- and concentration-dependent cytotoxicity, oxidative stress, DNA damage, impaired mitochondrial respiration, and metabolic dysregulation. Topical exposure in mice increased cutaneous IL-6 and TNF-α levels and induced histopathological abnormalities involving skin appendages and adipose-tissue organization. These findings provide preliminary evidence of LCM-associated cutaneous toxicity, but the limited number of experimental studies and uncertainty regarding the relevance of the tested doses to realistic dermal exposure currently preclude firm conclusions regarding human skin toxicity (30).

### 4.7 Endocrine toxicity

The endocrine system represents another important target of LCM toxicity, and current studies have provided relatively consistent evidence from two major aspects: nuclear receptor perturbation and thyroid hormone disruption (28, 29, 34). Zhao et al. (28) reported that some LCMs exhibit potential PPARγ antagonistic activity and markedly disturb fatty acid β-oxidation and related metabolic networks. Given the central role of PPARγ in lipid metabolism, energy homeostasis, and cellular differentiation, the available evidence suggests that LCMs may affect endocrine and metabolic balance through receptor-mediated mechanisms (28).

Heldman et al. (34) conducted an *in vitro* screening of ten commercially available high-priority LCMs, comprising five nonfluorinated and five fluorinated LCMs, as well as three fluorination-based mixtures: the LCM-ALL mixture containing all ten LCMs, the LCM-NF mixture containing the five nonfluorinated LCMs, and the LCM-F mixture containing the five fluorinated LCMs. The study found that both individual monomers and mixtures altered the transcriptional activity of one or more nuclear receptors (34). Notably, six LCMs and all mixtures antagonized the progesterone receptor, and multiple LCMs as well as their mixtures promoted triglyceride accumulation and adipogenesis in murine preadipocytes and human mesenchymal stem cells (34). More importantly, the adipogenic effects predicted by the concentration addition model underestimated the observed effects, suggesting potential synergistic interactions under mixture exposure (34). These findings demonstrate nuclear-receptor perturbation and adipogenic responses to individual LCMs and defined mixtures in experimental models; however, the relevance of the tested mixture compositions and concentrations to real-world exposure remains uncertain.

In addition, He et al. (29) demonstrated in zebrafish and GH3 cell models that six common FLCMs disrupted thyroid hormone levels, affected key enzymes and proteins associated with the hypothalamic-pituitary-thyroid (HPT) axis, and exhibited clear thyroid hormone-disrupting activity. Molecular docking further suggested that these compounds may interact with thyroxine-binding globulin, integrin, and thyroid hormone receptor β (29).

These findings collectively support the potential of LCMs to interfere with nuclear receptor and thyroid hormone signaling. However, the evidentiary strength differs across experimental systems: receptor-based and cell-based assays provide mechanistic evidence, whereas zebrafish studies provide organism-level evidence of endocrine disruption; molecular docking, by contrast, is primarily predictive and cannot establish receptor-mediated effects *in vivo*. The extent to which these experimentally observed endocrine alterations occur at environmentally relevant human exposures, and whether they translate into clinically meaningful endocrine dysfunction, remains uncertain.

### 4.8 Metabolic toxicity and metabolic reprogramming

Metabolic toxicity has emerged as a recurring, cross-organ feature of LCM-induced toxicity (28, 32, 34, 49, 50). Rather than being confined to a single target tissue, metabolic disruption has been reported across hepatic, renal, endocrine, intestinal, developmental, and computational toxicology studies, suggesting that altered energy metabolism, lipid homeostasis, mitochondrial function, and nuclear receptor signaling may represent central mechanisms linking otherwise separate toxicological endpoints.

Evidence from organ-specific studies supports this interpretation. In the liver, EDPrB exposure in adult zebrafish induced disturbances in lipid and glucose metabolism, together with oxidative stress, endoplasmic reticulum stress, and apoptosis-related responses (32). In renal tubular epithelial cells, LCM exposure was associated with transcriptomic and metabolomic alterations, with fatty acid β-oxidation and stress-response pathways being prominently affected (28). Endocrine-related studies further indicate that LCMs can perturb PPARγ-mediated signaling, promote triglyceride accumulation, and induce adipogenic differentiation, suggesting that metabolic toxicity may be mediated in part through nuclear receptor disruption and altered lipid-regulatory pathways (34).

Recent transcriptomic and computational studies further support metabolic toxicity as an important priority in LCM research. Zhao et al. (49) integrated RNA sequencing with a deep learning-based metabolic toxicity prediction framework to screen and prioritize LCMs with potential metabolic toxicity. Using six fluorinated LCMs with strong enrichment characteristics in zebrafish, the study found that metabolic disturbance was the predominant transcriptomic response, involving 28 KEGG pathway abnormalities and 498 differentially expressed genes (49). The authors then developed the LCMsT-MTP model, which integrates LCM structural information with metabolic toxicity target sequences to predict 21 metabolic toxicity endpoints across a broader set of LCMs (49). This work extends metabolic toxicity assessment from experimental transcriptomic observation to risk-oriented computational prioritization, although the predicted toxicity rankings should still be interpreted as screening-level evidence that requires further experimental validation (49).

Consistent with this mechanistic direction, Wang et al. investigated mitochondrial energy metabolism in zebrafish embryos and larvae exposed to five representative FLCMs, including EDPrB, DTMDPB, TPrCB, EDPB, and EDFPBB (50). Exposure across 0.05–50 μg/L, a range that included concentrations considered environmentally relevant in the original study, reduced basal respiration, ATP-linked oxygen consumption, NAD^+^/NADH ratio, and mitochondrial membrane potential, indicating impaired oxidative phosphorylation and mitochondrial bioenergetic dysfunction (50). The study further showed compensatory activation of TCA-cycle-related enzymes and AMPK-associated rerouting of carbon flux toward amino acid-driven anaplerosis (50). Pharmacological inhibition with Compound C provided additional support for AMPK involvement, positioning the AMPK–glutamate–TCA axis as a key adaptive pathway in FLCM-induced metabolic reprogramming (50).

Taken together, the experimental and computational evidence points to metabolic disturbance as a recurring feature of LCM toxicity. However, these lines of evidence differ substantially in evidentiary strength. Experimental transcriptomic, biochemical, and mitochondrial findings provide direct evidence of biological responses in specific models, whereas computational predictions primarily support chemical prioritization and mechanistic hypothesis generation. In addition, the available studies do not yet establish congener-specific dose–response relationships or clarify whether the observed metabolic alterations persist under chronic, environmentally relevant exposure in humans. The broader contribution of metabolic reprogramming to long-term human disease therefore remains to be determined.

### 4.9 Reproductive toxicity

Current evidence supports maternal–fetal exposure to LCMs and suggests that they may pose reproductive risks through placental transfer and placental dysfunction (22). Using an integrated approach that combined human biomonitoring, uterine perfusion in pregnant rats, and placental cell experiments, Zhang et al. (22) found that 14 of 56 target LCMs were detected in both maternal serum and cord serum. This finding suggests that LCM exposure extends beyond environmental presence and can result in maternal internal exposure with subsequent transfer to the fetal circulation (22). Further analyses in the same study suggested that SLC16A10 may contribute to the transplacental transport of certain LCMs. This conclusion was supported not only by transporter expression analysis but also by cell-based siRNA knockdown experiments, which provided functional evidence for the involvement of SLC16A10 in LCM transport. Nevertheless, the transporter specificity, substrate selectivity among different LCM congeners, and *in vivo* contribution of SLC16A10 to placental transfer remain to be further confirmed. The same study further suggested that exposure to these LCMs was associated with impaired placental development and reduced progesterone release (22). Given the central role of the placenta in maternal–fetal exchange and pregnancy maintenance, these findings suggest that LCMs may impair placental function and thereby disturb maternal–fetal homeostasis (22). In addition, Wu et al. reported that long-term exposure to the representative liquid crystal monomer 4-cyano-4′-ethoxybiphenyl (EBCN) at concentrations described by the authors as environmentally relevant induced testicular dysfunction in mice (36). EBCN exposure was associated with reduced sperm count, abnormal sperm morphology, reduced numbers of Leydig and spermatogenic cells, disruption of testosterone/LH/FSH homeostasis, gut microbial dysbiosis, reduced production of the anti-inflammatory metabolite L-carnosine, elevated serum inflammatory cytokines, and activation of necroptosis-related pathways (36). Together, these findings suggest that gut microbial dysbiosis and systemic inflammation may contribute to EBCN-induced testicular injury through the gut–testis axis (36).

However, the current evidence is still largely limited to placental transfer, placental dysfunction, and a small number of experimental observations of male reproductive injury. Systematic evidence on fertility, spermatogenesis, reproductive hormone profiles, pregnancy outcomes, and postnatal reproductive effects remains scarce, particularly in human populations. Thus, although LCMs represent a potential reproductive health concern, the magnitude, dose dependence, and human relevance of these effects remain insufficiently defined.

### 4.10 Developmental toxicity

Available studies suggest that LCMs have developmental toxic potential, and their effects may be manifested not only as impaired placental support for development, but also as abnormalities in neural and sensory system development during embryonic or larval stages (22).

One line of evidence comes from the study by Zhang et al. (22), which suggested that certain LCMs may undergo transplacental transfer and may be associated with impaired placental development and reduced progesterone release, potentially compromising the intrauterine environment required for normal fetal development. In this context, the placenta should be considered not only a route of exposure transfer, but also one of the key target sites involved in LCM-induced developmental toxicity (22). Although the maternal–fetal biomonitoring findings provide evidence of developmental exposure to LCMs, they do not by themselves establish adverse developmental outcomes. The accompanying placental experiments provide biological support for impaired placental function, but the extent to which these findings translate into clinically relevant developmental toxicity in humans remains uncertain.

Another body of evidence comes from He et al. (29), who found that FLCMs disrupted thyroid hormone signaling, damaged retinal structure, and significantly suppressed visual motor responses. Given the critical role of thyroid hormones in nervous system development and sensory-organ maturation, evidence supports the possibility that LCMs may induce developmental toxicity through an endocrine-disruption-mediated neurodevelopmental pathway (29). These findings provide experimental evidence linking FLCM exposure to developmental and sensory abnormalities and support a plausible endocrine-mediated mechanism. However, the evidence is derived primarily from zebrafish and experimental developmental models, and species-specific differences in thyroid physiology, toxicokinetics, and developmental sensitivity may limit direct extrapolation to humans. Thus, these findings support developmental hazard and mechanistic plausibility, but their relevance to human developmental outcomes remains to be established.

Taken together, the available experimental findings suggest that developmental effects of LCMs may extend beyond gross morphological abnormalities to include alterations in sensory and neurobehavioral function. However, whether such effects persist over longer developmental periods or translate into adverse neurodevelopmental outcomes in humans remains unclear. Overall, the current evidence supports developmental hazard and biological plausibility, but the magnitude and human relevance of LCM-related developmental toxicity remain insufficiently defined.

The major experimental toxicological and toxicokinetic evidence discussed above is summarized in Supplementary Table S3.

### 4.11 Preliminary structure–toxicity hypotheses for liquid crystal monomers

Drawing on the system- and tissue-specific evidence reviewed above, several tentative structure–toxicity patterns can be discerned across the currently available LCM studies. These patterns do not establish a quantitative or semi-quantitative structure–activity relationship (SAR) framework, because most studies have examined only a few monomers using different experimental models and endpoints. Nevertheless, the available evidence suggests that fluorination pattern, cyano substitution, alkyl or alkoxy chain length, hydrophobicity, and aromatic backbone rigidity may influence tissue distribution, target-organ preference, and toxicological profiles. The key structural characteristics and preliminary structure–toxicity patterns based on recent toxicological evidence are summarized in Table 2. Accordingly, the apparent structure–toxicity relationships should be interpreted in the context of the underlying experimental models and exposure conditions rather than as direct evidence of class-wide differences in toxic potency.

**Table 2:** Compound-specific observations and preliminary structure–toxicity hypotheses for LCMs based on studies published since 2023.

Structural feature or study group	Representative compounds	Experimental observations and mechanistic findings	Preliminary structure–toxicity hypothesis	Evidence limitation	References
Selected fluorinated LCMs	DFECB; EDPrB	**Observed effects**: DFECB exposure induced renal dysfunction in mice, whereas EDPrB exposure induced hepatic injury in adult zebrafish. **Mechanistic findings**: DFECB-associated renal effects involved disruption of PPARα-mediated fatty acid oxidation; EDPrB exposure was accompanied by oxidative stress and metabolic alterations.	Fluorination may influence receptor interactions and metabolic toxicity, but its independent contribution has not been established.	The compounds were examined in different experimental models. Matched fluorinated versus nonfluorinated comparisons and standardized potency assessments are lacking.	(32, 33)
Selected cyano-substituted LCMs	EBCN; EtCBN	**Observed effects**: EBCN exposure impaired intestinal epithelial function in organoid experiments and testicular function in mice. EtCBN exposure impaired cognitive function in mice. **Mechanistic findings**: Mouse studies reported gut microbiota alterations and inflammatory responses associated with the effects of these compounds.	Cyano substitution may be relevant to the reported gut–target-organ effects, but its independent contribution cannot be determined from the available compound-specific studies.	Controlled comparisons with structurally related non-cyano analogs are unavailable.	(35–37)
LCMs showing tissue-specific distribution	Four LCMs (3dFB, 2OdF3B, 2teFT, 6OCB)	Toxicokinetic observations: The tested compounds distributed to lipid-rich tissues, including the liver, adipose tissue, and brain, in mice. Tissue distribution alone does not establish toxicity in these organs.	Hydrophobicity may contribute to tissue partitioning.	No quantitative relationship between consistently measured logKow values and tissue burdens was established.	(27)
Fluorinated and nonfluorinated LCM mixtures	Ten LCMs and three mixtures	**Observed cellular effects**: Individual LCMs and mixtures promoted lipid accumulation and adipogenesis *in vitro*. **Mechanistic findings**: Receptor-based assays demonstrated altered nuclear-receptor activity. These findings do not by themselves establish adverse metabolic outcomes in humans.	Toxicological activity may depend on both individual structure and mixture composition.	Evidence is mainly based on *in vitro* assays and does not isolate the independent effect of fluorination.	(34)

Experimental observations refer to findings measured in the specified compounds and models and do not establish adverse effects in humans. Mechanistic findings are distinguished from organ-level or functional adverse effects; associations alone do not demonstrate causal mediation. Tissue-distribution findings represent toxicokinetic evidence rather than direct evidence of organ toxicity. The proposed structure–toxicity relationships are qualitative and hypothesis-generating because matched homologous comparisons and standardized cross-compound potency assessments are lacking. No formal quantitative structure–activity relationship (QSAR) model was developed in this review.

At present, the available evidence supports compound-specific observations rather than generalizable structural determinants of toxicity. DFECB and EDPrB, two fluorinated LCMs, have been associated with renal and hepatic metabolic injury, respectively, whereas the cyano-substituted compounds EBCN and EtCBN have been linked to gut microbiota disturbance, intestinal injury, reproductive toxicity, neuroinflammation, and cognitive impairment. Tissue-distribution studies further suggest that hydrophobicity may contribute to partitioning into lipid-rich tissues. However, these studies did not use matched homologous compounds under identical experimental conditions, and consistent logKow values, standardized potency estimates, and controlled comparisons of fluorination position, fluorine number, cyano substitution, or alkyl/alkoxy chain length are generally unavailable. These apparent patterns should therefore be considered preliminary hypotheses rather than established SAR conclusions (27, 32, 33, 35–37).

Overall, research on structure–toxicity relationships of LCMs remains at an early, largely pattern-recognition stage. Although several structural features have been tentatively linked to elevated toxicological risk, systematic validation using homologous compounds, quantitative structure-related parameters, and rigorously controlled experimental systems is still lacking. Future studies should therefore integrate physicochemical descriptors, toxicokinetic information, and standardized toxicological endpoints to build a more robust and predictive SAR framework for LCMs.

## 5 Immune-relevant mechanisms and biological plausibility

Direct evidence for classical LCM-induced immunotoxicity remains limited because immune-cell phenotypes, immune-organ pathology, host-defense capacity, and functional immune endpoints have rarely been evaluated. Accordingly, the available evidence is more appropriately interpreted in terms of immune-relevant biological mechanisms and mechanistic plausibility rather than established immunotoxic effects.

For the purposes of this review, “classical immunotoxicity” refers to directly demonstrated adverse effects on immune-cell phenotypes, immune organs, host-defense functions, or other validated functional immune endpoints, whereas “immune-relevant mechanisms” refers to biological processes that may influence immune regulation or inflammatory homeostasis without, by themselves, establishing functional immune impairment.

Current findings can be grouped into three interconnected mechanistic domains: oxidative stress and inflammatory signaling, endocrine–metabolic–immune crosstalk, and gut microbiota or barrier dysfunction. These pathways may help explain convergent effects observed across organ systems, but their translation into functional immune impairment requires direct experimental validation.

### 5.1 Critical appraisal of the evidence base

The evidence linking LCM exposure to immune-relevant effects is heterogeneous in both study design and evidentiary strength. Human biomonitoring studies provide the most direct evidence of internal exposure and are therefore particularly important for establishing human relevance. However, the available studies are predominantly cross-sectional and provide limited information on exposure history, temporal variability, and causal relationships. Animal studies offer more direct evidence of systemic toxicity, tissue distribution, and biological responses under controlled exposure conditions, but extrapolation to humans remains subject to interspecies differences in toxicokinetics, metabolism, exposure route, and dose. Cellular models are useful for defining direct molecular and cellular responses, whereas organoid systems can provide greater tissue-level relevance; nevertheless, neither model fully captures the integrated toxicokinetic, endocrine, metabolic, and immune interactions that occur *in vivo*.

Computational approaches, including molecular docking, adverse outcome pathway-based prediction, and machine-learning models, provide valuable tools for identifying potential molecular targets and prioritizing compounds for further investigation. However, these approaches are inherently dependent on the quality and biological relevance of the underlying data and should not be regarded as direct evidence of toxic effects. In particular, molecular docking can suggest structural compatibility between a compound and a putative target, but docking results alone cannot establish direct binding, functional modulation, or causal pathway involvement. Computational predictions were therefore interpreted as hypothesis-generating or screening-level evidence, particularly when independent experimental validation was limited. Overall, greater confidence was placed in findings supported by convergent evidence from complementary experimental systems, whereas findings derived from a single model or predictive approach were interpreted more cautiously.

This distinction is particularly relevant to the interpretation of immune-related effects. Current studies consistently point to oxidative stress, inflammatory signaling, endocrine–immune interactions, gut barrier dysfunction, and microbiota alterations as processes that may influence immune regulation. However, direct evidence of classical immunotoxicity, including alterations in immune-cell populations, immune-organ pathology, host-defense function, or immune memory, remains limited. Thus, the available evidence provides biological plausibility for immune-related effects and helps define mechanistic hypotheses, but it does not yet establish LCMs as confirmed immunotoxicants.

### 5.2 Oxidative stress and inflammatory signaling

Oxidative stress and inflammation-related signaling represent one of the most consistently reported immune-relevant mechanistic domains in current LCM studies. These processes should be interpreted as upstream mechanisms relevant to immune regulation rather than as direct evidence of immune injury. Previous studies have shown that various LCMs can significantly increase intracellular reactive oxygen species (ROS) levels *in vitro*, accompanied by impaired antioxidant defense, reduced cell viability, and metabolic disturbance. Transcriptomic and metabolomic analyses have further shown that LCM exposure perturbs inflammation- and stress-related signaling networks, including NF-κB- and TNF-associated pathways (28, 33). Collectively, these findings indicate that, although LCMs have not yet been adequately validated in classical immune cell models, they provide mechanistic plausibility for inflammatory amplification and potential perturbation of immune-regulatory pathways (28, 32, 33).

### 5.3 Endocrine–metabolic–immune crosstalk

Endocrine–immune crosstalk may represent a plausible pathway through which LCMs could influence immune-relevant regulation. However, whether these endocrine and metabolic alterations lead to measurable immune dysfunction remains to be directly tested. Zhao et al. (28) reported that some LCMs may act as potential antagonists of peroxisome proliferator-activated receptor gamma (PPARγ), thereby disrupting lipid metabolism and related transcriptional regulatory processes. Subsequently, Peng et al. found that DFECB, a representative FLCM, could induce renal metabolic injury by interfering with PPARα-mediated fatty acid oxidation, further supporting the metabolic disruption potential of LCMs (33). In addition, Heldman et al. (34) demonstrated *in vitro* that individual monomers and their mixtures could alter multiple nuclear receptor signaling pathways and promote lipid accumulation, indicating broader endocrine-disrupting effects (34). More directly relevant to immune-related hazard assessment, Cheng et al. (31) used a machine-learning-assisted framework to prioritize LCMs with potential immune-related hazards and to nominate candidate molecular targets (31). Among these, 11β-hydroxysteroid dehydrogenase 1 (11β-HSD1/HSD11B1) emerged as a candidate target with potential relevance to endocrine–immune regulation. Molecular docking and additional mechanistic analyses were subsequently used to explore this possibility. However, the computational and docking components of the study should be interpreted primarily as target-prioritization and hypothesis-generating evidence rather than as proof of direct molecular binding, functional modulation of 11β-HSD1, causal pathway involvement, or immune dysfunction *in vivo*. Because 11β-HSD1 participates in local glucocorticoid metabolism and inflammatory regulation, its predicted involvement provides a biologically plausible basis for further investigation. Whether LCM exposure alters 11β-HSD1 activity to an extent sufficient to affect glucocorticoid signaling or immune regulation remains to be established. Accordingly, 11β-HSD1 should currently be regarded as a candidate mechanistic target requiring further biochemical, cellular, and *in vivo* validation rather than as an established mediator of LCM-induced immunotoxicity.

### 5.4 Gut microbiota, barrier dysfunction, and immune-relevant regulation

Gut microbiota dysbiosis and disruption of the gut–target organ axis provide emerging evidence relevant to the immune-related effects of LCMs. Wu et al. (36) found in mice that long-term exposure to the representative liquid crystal monomer EBCN at concentrations described by the authors as environmentally relevant reduced gut microbiota diversity, decreased the anti-inflammatory metabolite L-carnosine, and was accompanied by elevated serum inflammatory factors, suggesting that gut microecological disruption may contribute to systemic inflammatory imbalance and remote target-organ effects. The reproductive outcome reported in this study is discussed in Section 4.9. In addition, Zhang et al. (35), using intestinal organoids and the adverse outcome pathway (AOP) framework, showed that LCM mixtures and EBCN could inhibit organoid expansion, damage the intestinal barrier, and disturb intestinal stem cell homeostasis, with complement component 3 (C3) proposed as a potential molecular initiating event for downstream immune-relevant effects. The organoid findings provide useful tissue-level evidence for barrier and epithelial responses, but they do not fully reproduce systemic toxicokinetics, circulating immune-cell interactions, or whole-organism endocrine and metabolic regulation. These findings suggest that LCM-induced intestinal barrier disruption, microecological imbalance, and abnormal inflammatory mediator signaling may have systemic immune-relevant implications beyond the intestine (35, 36).

Collectively, the available evidence indicates that immune-related effects of LCMs remain an emerging area of investigation. While multiple mechanistic pathways converge on processes relevant to immune regulation, direct experimental validation in classical immunotoxicological models is still lacking. Consequently, current findings should be viewed as providing biological plausibility and research hypotheses rather than establishing immunotoxicity as a confirmed toxicological endpoint.

## 6 Discussion

### 6.1 Qualitative health-risk considerations

The available evidence remains insufficient to support a comprehensive quantitative assessment of human health risks across the LCM class. To clarify the contribution and limitations of the available evidence, the following discussion distinguishes hazard identification, exposure assessment, dose–response evidence, and risk characterization, with uncertainties summarized separately. Therefore, the health-risk discussion in this review should be interpreted as a qualitative synthesis of risk-related evidence rather than as a quantitative risk assessment.

**Hazard identification**. Experimental studies suggest multisystem toxicity involving nervous, hepatic, renal, intestinal, respiratory, cutaneous, metabolic, endocrine, reproductive, and developmental endpoints, as summarized in Section 4. These findings identify potential hazards associated with the compounds and experimental conditions investigated, but do not establish equivalent toxicity across the entire LCM class or adverse effects at observed human exposure levels. As discussed in Section 5, oxidative stress, inflammatory signaling, and other immune-relevant mechanisms provide biological plausibility, whereas direct evidence of classical immunotoxicity remains limited.

**Exposure assessment**. LCMs have been detected in human biological matrices, including urine, serum, human milk, and paired maternal and cord serum, documenting internal exposure in occupational and general-population settings and during sensitive life stages (18–25, 47, 48). Potential environmental exposure routes include dust ingestion, inhalation of particle-bound contaminants, dermal contact, and dietary intake, including infant formula (13, 16, 17, 51). Maternal–fetal and lactational transfer may then occur after maternal internal exposure. Animal tissue-distribution studies provide complementary evidence that some LCMs can accumulate in lipid-rich tissues and reach brain tissue (26, 27). However, biomonitoring measurements alone do not establish exposure frequency, duration, predominant sources, or target-tissue dose, and animal distribution findings cannot be directly extrapolated to humans.

**Dose–response evidence**. Selected experimental studies provide concentration- or dose-related observations, including time- and concentration-dependent cytotoxicity in keratinocytes exposed to an LCM mixture (30). However, the available evidence remains insufficient to establish robust congener-specific relationships for chronic low-dose exposure in humans. Nominal cell-culture concentrations, concentrations in zebrafish exposure water, administered animal doses, and measured human biomonitoring concentrations are not directly interchangeable. As discussed in Section 6.2, congener-specific toxicokinetic information and internal dosimetry are needed to relate experimental effect levels to human exposure. Future quantitative assessment should evaluate whether suitable studies support endpoint-specific points of departure and explicitly justify the extrapolation methods and uncertainty factors applied.

**Risk characterization**. Taken together, current evidence supports concern for possible health risks but is not sufficient to define broadly applicable safe exposure thresholds, estimate population-level disease burden, or reliably quantify the likelihood and severity of adverse effects at observed human exposure levels. Infants, pregnant women, and populations living or working near e-waste recycling areas should be considered priority populations for future risk assessment because of potentially elevated exposure, greater biological susceptibility, or both. Published risk estimates should be interpreted within the specific congeners, exposure routes, populations, toxicological benchmarks, and assumptions evaluated, rather than generalized to the entire LCM class or to aggregate exposure across routes and mixtures.

**Priority congeners**. The available evidence identifies several LCMs that deserve priority in monitoring and toxicological research. For exposure monitoring, BDPrB stands out because it was frequently detected in paired indoor dust and human-milk samples in Beijing and dominated the human-milk concentration profile (20). BDFPrCB is particularly relevant to prenatal exposure: in the Qingyuan study, it was detected in 100% of maternal serum samples and 98.9% of cord serum samples and had the highest median concentrations in both matrices (22). Concentration profiles also identify priorities specific to individual settings, including EtCBN and PBIPHCN in residential indoor dust, BEEB in serum and urine from Dingxi, EDPeB in human milk from Hunan, and MOPB in the national human-milk survey (16, 24, 25, 48). Monitoring targets should therefore be selected according to the population and sample matrix, taking differences in analytical coverage into account.

EBCN and EtCBN are priorities for further toxicological research because both have been detected in environmental or human samples and have shown adverse effects in experimental models. EBCN was a major contributor to urinary LCM concentrations in children, and experimental studies showed intestinal barrier damage and testicular injury involving disruption of the gut–testis axis (35, 36, 47). EtCBN was one of the predominant congeners in residential indoor dust and caused neuroinflammation and cognitive impairment in mice (16, 37). DFECB and EDPrB are also candidates for further testing because of their reported renal and hepatic effects, respectively, including disturbances in fatty acid oxidation and hepatic metabolism (32, 33). Together, these findings support closer investigation of these compounds across a wider range of doses, exposure durations, and biological endpoints.

These priorities reflect the available exposure and hazard evidence rather than an established ranking of human health risks. High detection frequency or concentration does not necessarily indicate greater toxicity, and frequently detected congeners with little toxicological information should remain a focus of research. Reliable comparisons of potency will require testing under comparable conditions and measurements of internal dose. Monitoring panels and testing priorities should therefore be updated as evidence becomes available for a broader range of LCM structures.

**Uncertainties**. Major uncertainties include incomplete congener coverage, heterogeneous exposure measurements, limited geographical representativeness, insufficient human toxicokinetic information, interspecies and model-to-human extrapolation, limited chronic low-dose evidence, and poorly characterized mixture interactions. These uncertainties constrain the integration of hazard, exposure, and dose–response evidence and limit confidence in quantitative risk estimates. The predominantly cross-sectional nature of human health-related studies and potential confounding further limit causal inference. These limitations are discussed in detail in Section 6.2. Robust future risk assessment will require standardized biomonitoring, congener-specific toxicokinetic data, long-term low-dose toxicity studies, mixture-effect evaluation, and toxicological endpoints that can be linked to adverse health outcomes.

### 6.2 Evidence gaps and review limitations

Although studies on the environmental occurrence, human exposure, and biological toxicity of LCMs have increased in recent years, this field remains at an early stage overall, and the existing evidence is still insufficient to support a comprehensive quantitative assessment of their environmental and human health risks (3).

The range of LCMs examined to date remains narrow, with most studies focusing on only a handful of representative compounds, whereas a wide variety of LCMs with diverse and complex structures are involved in their actual production and application processes. Differences in functional groups, substituents, and degrees of fluorination may confer markedly distinct environmental behaviors and toxic effects. Therefore, the representativeness of current findings for the entire class of LCM pollutants remains clearly limited (34).

The current evidence on structure–toxicity relationships remains largely hypothesis-generating. As discussed in Section 4.11, fluorination pattern, cyano substitution, alkyl or alkoxy chain length, hydrophobicity, and aromatic backbone rigidity may influence tissue distribution, target-organ preference, and toxicological profiles. However, these observations are derived from different compounds, experimental models, exposure conditions, and endpoints rather than from matched homologous series tested under standardized conditions. The lack of consistent physicochemical descriptors, standardized potency estimates, and controlled comparisons of individual structural features currently precludes the development of a robust structure–activity relationship framework for LCMs (27, 32, 33, 35–37).

Many toxicological studies rely on short-term *in vitro* or animal exposure designs, and some use doses that cannot be directly related to measured environmental or human exposure levels, limiting quantitative extrapolation to real-world low-dose, chronic exposure (10, 34). In addition, the environmental relevance of experimental exposure concentrations should be interpreted carefully across studies, because measured environmental levels are often reported in dust, air, or biological matrices, whereas toxicological assays use concentrations in culture medium or administered doses in animal models.

The available data illustrate this exposure–dose mismatch. Human biomonitoring studies reporting serum concentrations in ng/mL have reported median LCM concentrations ranging from 0.92 to 126.55 ng/mL across different study populations and analyte panels, whereas environmental measurements may be reported on a mass-of-matrix basis, such as 43.7–448 ng/g in ventilation and air-conditioning filter dust. Experimental studies, by contrast, have used exposure metrics such as 0.05–50 μg/L in zebrafish water, 0.34–34 μg/mL in cell-culture medium, and administered doses including 20–200 μg/kg for EtCBN and 10 mg/kg bw/day for DFECB in separate mouse studies. These values are not directly interchangeable because they represent different matrices and dose metrics, and the relationship between an external or administered dose and the resulting internal concentration depends on exposure route, duration, absorption, bioavailability, metabolism, congener-specific toxicokinetics, and tissue distribution. Without matched internal dosimetry or toxicokinetic bridging, current experimental findings should therefore be interpreted primarily as evidence of hazard and biological plausibility rather than as quantitative estimates of risk at observed human exposure levels.

Moreover, real-world LCM exposure is likely to involve mixtures, whereas most mechanistic studies have focused on individual compounds. The limited mixture studies available to date provide preliminary evidence that combined effects may differ from those inferred from single-compound testing. Reconstructed LCM mixtures induced oxidative and cytotoxic responses in BEAS-2B cells, a five-LCM mixture produced cutaneous toxicity in cellular and mouse models, and fluorination-based mixtures altered nuclear-receptor activity and adipogenesis; notably, concentration-addition predictions underestimated the observed adipogenic effects in one study (10, 30, 34). An intestinal organoid study also reported impaired organoid expansion and abnormal crypt budding following exposure to LCM mixtures (35). However, mixture composition, component ratios, exposure metrics, and experimental systems differ substantially among studies, preventing firm conclusions regarding the magnitude and reproducibility of mixture interactions. More environmentally representative mixture designs and component-resolved analyses are therefore needed.

In addition, toxicokinetic evidence remains a major limitation in interpreting the human relevance of LCM toxicity. Human biomonitoring has demonstrated internal exposure and maternal–fetal transfer of selected LCM congeners, whereas experimental animal studies indicate systemic distribution and accumulation in the brain, liver, adipose tissue, and other lipid-rich compartments. However, congener-specific absorption, distribution, metabolism, excretion, barrier transport, and tissue-partitioning behavior remain insufficiently characterized, particularly in humans (3, 22, 26, 27, 37, 52). Human-milk measurements demonstrate lactational exposure but do not by themselves establish transfer efficiency because paired maternal serum–milk measurements and congener-specific partition information remain limited (20, 24, 25). These toxicokinetic gaps hinder the translation of external or administered doses into biologically relevant internal or target-tissue concentrations and therefore limit quantitative extrapolation between experimental models and human exposure.

Geographical coverage of the available exposure evidence is also uneven. Environmental occurrence and human biomonitoring studies summarized in this review are concentrated predominantly in China, with more recent environmental evidence from Pakistan, while corresponding data from many other geographic regions remain scarce (3, 18–25, 38). This concentration limits the generalizability of current exposure estimates and constrains assessment of geographic variability in LCM contamination and human exposure. Broader multi-region environmental monitoring and human biomonitoring studies using harmonized analyte panels and analytical methods are therefore needed to determine whether the exposure patterns currently reported are representative of populations outside the regions studied.

This review also has several limitations. Although a structured database search was conducted using predefined eligibility criteria, the review was intended as a critical narrative synthesis rather than a systematic review. Accordingly, the protocol was not prospectively registered, and no formal study-level risk-of-bias tool was applied. Instead, the evidentiary strength and major limitations of the included studies were appraised qualitatively according to study design, exposure relevance, model relevance, exposure dose and duration, endpoint specificity, consistency across studies, replication, and experimental validation. Restricting the search to peer-reviewed articles published in English may also have resulted in the omission of relevant evidence. Given the substantial heterogeneity across the included studies, the conclusions should therefore be interpreted as a critical qualitative appraisal of the available evidence rather than as definitive causal or quantitative risk estimates.

### 6.3 Future research priorities

Future research should address four priorities. Compound selection should be guided by the monitoring and toxicological priorities outlined in Section 6.1. Targeted monitoring should cover frequently detected or abundant congeners, including BDPrB and BDFPrCB, and be complemented by high-resolution mass spectrometry and suspect screening to identify additional LCMs. Toxicological studies should extend the evidence for EBCN, EtCBN, DFECB, and EDPrB and address the lack of toxicity data for other prominent congeners in environmental and human samples. Comparisons of structurally related compounds under the same experimental conditions are needed to determine how fluorination, cyano substitution, and other structural features influence toxicokinetics and toxicity.

Toxicity assessment should more closely reflect real-world environmental exposure scenarios by strengthening studies on long-term chronic exposure at environmentally relevant doses. In addition, multi-organ cell models, organoid models, and animal experiments should be integrated to enhance the biological relevance of the findings and improve their value for risk extrapolation at multiple levels. Where feasible, future experimental studies should report achieved internal or tissue concentrations alongside nominal or administered doses and compare these values with human biomonitoring data to improve exposure–dose interpretation and cross-species extrapolation.

Future studies should routinely assess mixture exposure using designs that reflect the composition and relative concentrations of LCMs in environmental and human samples. At the same time, greater attention should be paid to sensitive populations and critical exposure windows. In particular, future research should prioritize large, adequately powered prospective mother–infant cohorts with repeated sampling during pregnancy, lactation, and early childhood. Such cohorts would better characterize critical exposure windows and the temporal variability of LCM exposure. Biomonitoring should ideally include maternal serum, cord blood, maternal and infant urine, and human milk. Follow-up assessments should cover birth outcomes, growth, neurodevelopment, endocrine and reproductive function, and immune-related biomarkers such as IL-6, TNF-α, and complement C3. Where feasible, these assessments should also include validated measures of immune function and indicators of placental function. Candidate molecular targets emerging from computational prioritization, including 11β-HSD1, should be independently validated using biochemical, cellular, and *in vivo* approaches before their mechanistic roles in LCM toxicity are considered established.

Greater emphasis should be placed on well-defined immune-related endpoints, metabolic toxicity, endocrine-disrupting effects, and toxicokinetic studies to systematically elucidate the internal transport, tissue distribution, target-organ accumulation, and molecular initiating events of LCMs *in vivo*. Future studies should further determine whether immune-relevant mechanistic alterations, such as oxidative stress, inflammatory responses, receptor-mediated signaling, and metabolic or epigenetic regulation, ultimately translate into adverse health outcomes.

## 7 Conclusion

LCMs are emerging contaminants detected in indoor and outdoor environmental media, with documented human exposure and a rapidly expanding toxicological evidence base. Their detection in serum, urine, human milk, maternal serum, and cord serum indicates exposure in the general population and during sensitive life stages. However, environmental detection and biomonitoring do not yet identify the predominant exposure sources, establish the persistence of internal exposure, or demonstrate causal associations with human disease. Experimental studies identify neurological, hepatic, renal, intestinal, respiratory, cutaneous, endocrine, reproductive, developmental, and metabolic effects, while oxidative stress, inflammatory signaling, nuclear receptor perturbation, metabolic dysregulation, and gut microbiota disruption provide plausible links across organ systems. However, direct evidence of classical immunotoxicity and human disease risk remains limited. Major gaps include incomplete toxicokinetic characterization, heterogeneous analyte coverage, insufficient chronic low-dose and mixture studies, and the absence of congener-specific reference values. Future research should combine harmonized biomonitoring and prospective studies in susceptible populations with environmentally relevant mixture testing, mechanistic validation, and assessment of functional immune endpoints. Compound selection should draw on the priorities identified here, including BDPrB and BDFPrCB for exposure monitoring and EBCN, EtCBN, DFECB, and EDPrB for further toxicological investigation. Such evidence is needed to move the field from hazard identification toward robust health-risk assessment and informed management of LCM exposure.
