# Supplementary material for: From environmental occurrence to human exposure: multisystem toxicity, immune-relevant mechanisms, and gaps in health-risk assessment of liquid crystal monomers
Source: Front Public Health. 2026 Oct 12;14:1977975. doi: 10.3389/fpubh.2026.1977975 (PMC13697307; doi:10.3389/fpubh.2026.1977975)
Supplement: Supplementary file 1 [file Table_1.docx]

**Supplementary Material**

**Supplementary Table S1. Complete database-specific search strategies for PubMed, Web of Science Core Collection, and Scopus**

Search date: 22 July 2026. All databases were searched from inception to the search date.

| Database | Search field | Filters applied | Records before filtering | Records retained | Search expression |
| --- | --- | --- | --- | --- | --- |
| PubMed | Title/Abstract and all searchable fields according to PubMed syntax | English language | 151 | 151 | ("liquid crystal monomer*"[tiab] OR "liquid-crystal monomer*"[tiab] OR "liquid crystalline monomer*"[tiab] OR "fluorinated liquid crystal monomer*"[tiab] OR "liquid crystal display chemical*"[tiab] OR "LCD chemical*"[tiab] OR "biphenyl liquid crystal*"[tiab] OR "cyclohexyl liquid crystal*"[tiab]) AND ((environment*[tiab] OR occurrence[tiab] OR contamination[tiab] OR distribution[tiab] OR source*[tiab] OR release[tiab] OR emission*[tiab] OR dust[tiab] OR "indoor air"[tiab] OR "outdoor air"[tiab] OR airborne[tiab] OR "particulate matter"[tiab] OR "PM2.5"[tiab] OR "PM10"[tiab] OR soil[tiab] OR sediment*[tiab] OR water[tiab] OR leachate[tiab] OR landfill[tiab] OR "electronic waste"[tiab] OR "e-waste"[tiab] OR recycl*[tiab] OR food[tiab] OR diet*[tiab] OR "infant formula"[tiab] OR "skin wipe*"[tiab] OR "hand wipe*"[tiab] OR exposure[tiab] OR "human exposure"[tiab] OR "internal exposure"[tiab] OR "body burden"[tiab] OR biomonitor*[tiab] OR urine[tiab] OR serum[tiab] OR "human milk"[tiab] OR "breast milk"[tiab] OR "cord blood"[tiab] OR "cord serum"[tiab] OR "maternal serum"[tiab] OR occupational[tiab] OR intake[tiab] OR ingestion[tiab] OR inhalation[tiab] OR dermal[tiab] OR transplacental[tiab] OR lactational[tiab]) OR (toxic*[tiab] OR "adverse effect*"[tiab] OR "health risk*"[tiab] OR "risk assessment"[tiab] OR oxidative[tiab] OR inflamm*[tiab] OR immun*[tiab] OR neurotoxic*[tiab] OR hepatotoxic*[tiab] OR nephrotoxic*[tiab] OR enterotoxic*[tiab] OR "respiratory toxic*"[tiab] OR "endocrine disrupt*"[tiab] OR "reproductive toxic*"[tiab] OR "developmental toxic*"[tiab] OR metabolic[tiab] OR toxicokinetic*[tiab] OR bioaccumulation[tiab] OR "tissue distribution"[tiab] OR "placental transfer"[tiab] OR "blood brain barrier"[tiab] OR "gut microbiota"[tiab] OR "intestinal barrier"[tiab] OR "nuclear receptor*"[tiab] OR PPAR[tiab] OR "thyroid hormone"[tiab] OR "complement C3"[tiab])) AND english[lang] |
| Web of Science Core Collection | Topic | Article; English language | 238 | 217 | ("liquid crystal monomer*" OR "liquid-crystal monomer*" OR "liquid crystalline monomer*" OR "fluorinated liquid crystal monomer*" OR "liquid crystal display chemical*" OR "LCD chemical*" OR "biphenyl liquid crystal*" OR "cyclohexyl liquid crystal*") AND ((environment* OR occurrence OR contamination OR distribution OR source* OR release OR emission* OR dust OR "indoor air" OR "outdoor air" OR airborne OR "particulate matter" OR "PM2.5" OR "PM10" OR soil OR sediment* OR water OR leachate OR landfill OR "electronic waste" OR "e-waste" OR recycl* OR food OR diet* OR "infant formula" OR "skin wipe*" OR "hand wipe*" OR exposure OR "human exposure" OR "internal exposure" OR "body burden" OR biomonitor* OR urine OR serum OR "human milk" OR "breast milk" OR "cord blood" OR "cord serum" OR "maternal serum" OR occupational OR intake OR ingestion OR inhalation OR dermal OR transplacental OR lactational) OR (toxic* OR "adverse effect*" OR "health risk*" OR "risk assessment" OR oxidative OR inflamm* OR immun* OR neurotoxic* OR hepatotoxic* OR nephrotoxic* OR enterotoxic* OR "respiratory toxic*" OR "endocrine disrupt*" OR "reproductive toxic*" OR "developmental toxic*" OR metabolic OR toxicokinetic* OR bioaccumulation OR "tissue distribution" OR "placental transfer" OR "blood brain barrier" OR "gut microbiota" OR "intestinal barrier" OR "nuclear receptor*" OR PPAR OR "thyroid hormone" OR "complement C3")) |
| Scopus | Title, abstract, and keywords | Article; English language | 272 | 227 | TITLE-ABS-KEY("liquid crystal monomer*" OR "liquid-crystal monomer*" OR "liquid crystalline monomer*" OR "fluorinated liquid crystal monomer*" OR "liquid crystal display chemical*" OR "biphenyl liquid crystal*" OR "cyclohexyl liquid crystal*") AND (TITLE-ABS-KEY(environment* OR occurrence OR contamination OR source* OR release OR emission* OR dust OR air OR airborne OR "particulate matter" OR "PM2.5" OR "PM10" OR soil OR sediment* OR water OR leachate OR landfill OR "electronic waste" OR "e-waste" OR recycl* OR food OR diet* OR "infant formula" OR exposure OR "human exposure" OR "internal exposure" OR biomonitor* OR urine OR serum OR "human milk" OR "breast milk" OR "cord blood" OR "cord serum" OR "maternal serum" OR intake OR ingestion OR inhalation OR dermal OR transplacental OR lactational) OR TITLE-ABS-KEY(toxic* OR "adverse effect*" OR "health risk*" OR "risk assessment" OR oxidative OR inflamm* OR immun* OR neurotoxic* OR hepatotoxic* OR nephrotoxic* OR enterotoxic* OR "respiratory toxic*" OR "endocrine disrupt*" OR "reproductive toxic*" OR "developmental toxic*" OR metabolic OR toxicokinetic* OR bioaccumulation OR "tissue distribution" OR "placental transfer" OR "blood brain barrier" OR "gut microbiota" OR "intestinal barrier" OR "nuclear receptor*" OR PPAR OR "thyroid hormone" OR "complement C3")) |
| Total |  |  | 661 | 595 |  |

Note: The database searches retrieved 661 records before application of the predefined language and article-type filters. After filtering, 151 records from PubMed, 217 records from the Web of Science Core Collection, and 227 records from Scopus were retained, yielding 595 records for export to EndNote 20. The PubMed language restriction was incorporated directly into the search expression. All 595 records were organized into database-specific groups in EndNote 20 and manually checked for duplication. A total of 323 duplicate records were removed, leaving 272 unique records for title and abstract screening.

**Supplementary Table S2. Expanded compound identities, exposure regimens, exposure-context information, and toxicological endpoints reported in studies of liquid crystal monomers**

| Evidence domain | Compounds | Expanded compound identity | Model | Exposure concentration | Exposure duration | Exposure relevance | Main findings and evidentiary role | Reference |
| --- | --- | --- | --- | --- | --- | --- | --- | --- |
| Neurotoxicity | Six FLCMs | Six common FLCMs: 2O3cHdFP [trans-1-ethoxy-2,3-difluoro-4-(4-propyl-cyclohexyl)-benzene; CAS: 174350-05-1]; tFPO-CF2-dF3B [4-[difluoro(3,4,5-trifluorophenoxy)methyl]-3,5-difluoro-4′-propyl-1,1′-biphenyl; CAS: 303186-20-1]; 2O3cHdFB [4-ethoxy-2,3-difluoro-4′-(trans-4-propylcyclohexyl)biphenyl; CAS: 189750-98-9]; 2OdFP3bcH [1-ethoxy-2,3-difluoro-4-[(trans,trans)-4′-propyl[1,1′-bicyclohexyl]-4-yl]-benzene; CAS: 123560-48-5]; TPrCB [1,2,3-trifluoro-5-[(trans,trans)-4′-propyl[1,1′-bicyclohexyl]-4-yl]benzene; CAS: 131819-23-3]; and BDPrB [trans-2,3-difluoro-4-(4-propylcyclohexyl)butoxybenzene; CAS: 208709-55-1]. | Danio rerio larvae | 2O3cHdFP: 3–300 ng/L; tFPO-CF2-dF3B: 1–100 ng/L; 2O3cHdFB: 0.45–45 ng/L; 2OdFP3bcH: 0.6–60 ng/L; TPrCB: 0.5–50 μg/L; BDPrB: 0.1–10 μg/L | 2–144 hpf | Exposure concentrations were set as low, medium, and high levels based on environmentally relevant concentrations and preliminary toxicity data; exposure solutions were renewed every 24 h. | E: Retinal structural disruption, reduced cell density in the ganglion cell layer, inner nuclear layer, and photoreceptor layer, and impaired visual motor response under red and green light. | (1) |
| Neurotoxicity | EtCBN | EtCBN [4-(4-ethylcyclohexyl)benzonitrile]. | Male C57BL/6J mice | 0, 20, and 200 µg/kg | 56 days | Oral gavage exposure model. The 20 μg/kg dose was designated as an environmentally relevant dose based on indoor dust occurrence of EtCBN and dust-ingestion-based exposure calculation. EtCBN was reported in indoor dust at ND–58.2 ng/g. | E: Cognitive impairment, anxiety-like behavior, impaired learning and memory, hippocampal edema and histopathological injury, reduced dendritic spine density, impaired hippocampal synaptic plasticity. M: Neuroinflammation, gut microbiota dysbiosis, reduced Muribaculum abundance, altered plasma metabolites, and activation of the NOD-like receptor signaling pathway. | (2) |
| Neurotoxicity | EtCBN | EtCBN [4-(4-ethylcyclohexyl)benzonitrile]. | BEND3 cells; BV2 cells; HT22 cells | 0.3, 3, and 30 µg/mL | 24 and 48 h | *In vitro* cytotoxicity model. The dose range was selected with reference to reported human serum LCM concentrations and prior *in vitro* LCM exposure design; the authors noted that *in vitro* tests directly evaluate cytotoxicity, whereas *in vivo* tests simulate environmental exposure. | E: Direct cytotoxicity in brain endothelial, microglial, and hippocampal neuronal cell lines; increased LDH release; BEND3 cells showed toxicity at 3 and 30 μg/mL within 24 h, BV2 cells showed cytotoxicity after 48 h exposure, and HT22 cells showed increased LDH release particularly at higher/prolonged exposure. | (2) |
| Toxicokinetics | Four target LCMs | Four target LCMs: 3dFB [3,4-difluoro-4′-propyl-1,1′-biphenyl; CAS: 118164-49-1]; 2OdF3B [1-ethoxy-2,3-difluoro-4-(4-propylphenyl)benzene; CAS: 157248-24-3]; 2teFT [4′′-ethyl-2′,3,4,5-tetrafluoro-1,1′:4′,1′′-terphenyl; CAS: 326894-55-7]; and 6OCB [4′-(hexyloxy)-4-biphenylcarbonitrile; CAS: 41424-11-7]. | Female BALB/c mice | 10 mg/kg bw | 0.5, 1, 3, 6, 24, 72, 168, and 336 h | Toxicokinetic tissue-distribution model after oral gavage; designed to characterize mammalian uptake, distribution, and tissue accumulation rather than to directly simulate environmental exposure. | TK: All four LCMs were detected in brain tissue after exposure, indicating blood–brain barrier penetration and brain distribution; brain half-lives were relatively long. Tissue accumulation alone does not establish neurotoxicity. | (3) |
| Toxicokinetics | Thirty-nine target LCMs | Mixture of thirty-nine target LCMs, including twenty-four fluorinated LCMs, six cyano-substituted LCMs, and nine alkyl-substituted LCMs: 3VbcH, MeO3bcH, Pe3bcH, 2O3cHdFP, 5MeB, 2OdF3B, tFMeO-2cHB, MePVbcH, tFPO-CF2-dF3B, MeP3bcH, tFMePO-CF2-dF3B, MeOdFP3bcH, 3cH2B, 2O2cHdFB, 2OdFP3bcH, 2F3T, 2O3cHdFB, 3OdFP3bcH, 2OdFP4bcH, 2F4T, 2bcHdFB, tFMePO-CF2-dF3PyB, tFMeO-3cHtFT, tFPO-CF2-tF3T, tFPO-CF2-dF3PyB, 3bcHdFB, 4bcHdFB, 5bcHdFB, 3dFB, 2CB, 3OCB, tFMeO-3bcHP, 2teFT, 5OCB, 6OCB, 8OCB, 5CT, b3cHB, and 3cH5cHB. | Adult zebrafish (Danio rerio) | 10 μg/L mixture | 19 days exposure followed by 12 days depuration | Bioaccumulation and depuration model in adult zebrafish; the exposure concentration was selected as 1/20 of the preliminary 96-h exposure concentration that caused no mortality. | TK: Tissue-specific bioaccumulation; Σ39LCM concentrations followed the order intestine > brain > gill > liver > muscle, indicating notable brain accumulation and bioaccumulation potential. | (4) |
| Hepatotoxicity | EDPrB | EDPrB [1-ethoxy-2,3-difluoro-4-(trans-4-propylcyclohexyl)benzene]. | Adult male zebrafish (Danio rerio) | 0, 1, 10, and 100 µg/L | 21 days | Environmentally relevant aqueous exposure model. Concentrations were selected based on reported EDPrB levels in aquatic environments and human serum; 100 μg/L was set as the highest chronic exposure concentration after no zebrafish mortality was observed at 10 mg/L within 96 h. | E: Hepatic morphological abnormalities, increased AST and ALT activities, oxidative stress, disruption of lipid and glucose metabolism. M: ER stress associated with PERK-eIF2α pathway activation, and apoptosis-related P53-Bax/Bcl2 signaling. | (5) |
| Toxicokinetics | Four target LCMs | Same four target LCMs as listed above. | Female BALB/c mice | 10 mg/kg bw for each compound, oral gavage | 0.5, 1, 3, 6, 24, 72, 168, and 336 h | Tissue-distribution and bioaccumulation model; useful for identifying target tissues relevant to biomonitoring and health-risk assessment. | TK: Preferential distribution to lipophilic tissues and organs, especially liver and adipose tissue; liver was a major distribution site, with mass contribution of 30%–59% at 6 h. | (3) |
| Toxicokinetics | Four target LCMs | Same four target LCMs as listed above. | Rat liver microsomes (RLM) | Initial concentration: 20 μM for each compound | 0, 15, 30, 60, 180, and 360 min | *In vitro* hepatic metabolism assay used to evaluate metabolic stability of LCMs in liver microsomes. | TK: Differential metabolic stability in RLM; 3dFB and 2OdF3B were rapidly metabolized, whereas 2teFT and 6OCB were more resistant to degradation. Stability ranking: 2teFT > 6OCB > 2OdF3B > 3dFB. | (3) |
| Nephrotoxicity | DFECB | DFECB [3,4-difluoro-4′-(trans-4-ethylcyclohexyl)biphenyl; CAS: 134412-18-3] | Female C57BL/6 mice, n = 9/group; oral gavage | 10 mg/kg bw/day | 30 days | The dose was selected following a preliminary dose-ranging experiment using 0.1–100 mg/kg bw/day. Exposure to 10 mg/kg bw/day produced a mean serum DFECB concentration of approximately 1.44 ng/mL, comparable to that reported in occupationally exposed workers. | E: Increased serum BUN and creatinine; renal interstitial inflammation and glomerular morphological alterations; M: Increased TNF-α and IL-6 expression; disrupted renal metabolic profiles; reduced PPARα expression and inhibition of fatty acid β-oxidation. | (6) |
| Nephrotoxicity | DFECB | Same compound as above. | Human renal proximal tubular epithelial cells (HK-2 cells); *in silico* molecular docking with PPARα | Cytotoxicity and ROS screening: 1 nM–500 μM; inflammatory assays: 1, 10, and 100 μM; ATP and NAD⁺/NADH assays: 10, 100, and 200 μM; PPARα-agonist rescue assay: 100 μM | 24 h for cell assays; not applicable for molecular docking | The concentration range included reported human internal-exposure levels and higher concentrations used for mechanistic evaluation; culture-medium concentrations are not directly equivalent to renal tissue exposure in humans. | E: Dose-dependent ROS generation and cytotoxicity; M: Increased TNF-α and IL-6; reduced ATP and NAD⁺/NADH ratio; downregulation of FAO-related genes; partial restoration of CPT1, CPT2, and ACSL1 expression by the PPARα agonist GW7647. H: Molecular docking suggested a possible DFECB–PPARα interaction involving hydrophobic and halogen contacts; direct binding was not established by docking alone. | (6) |
| Nephrotoxicity | Forty-four LCMs | Forty-four LCMs were investigated for toxicological screening; thirty-eight LCMs were used for inverse virtual screening, and three representative LCMs were selected for *in vitro* omics analysis. LCM 41 was identified as 4′-(octyloxy)-4-biphenylcarbonitrile (CAS: 52364-73-5). | Human kidney epithelial cells (HK-2 cells) | LCM 29 and LCM 41: 100 μM; LCM 7: 20 μM | 24 h | *In vitro* omics-based toxicity model used to evaluate cellular metabolic and transcriptomic responses to selected LCMs; direct comparison with environmental or human internal exposure levels remains limited. | M: Global metabolome and transcriptome dysregulation in HK-2 cells, including disruption of fatty acid β-oxidation, glycolysis, energy metabolism, oxidative stress response, lipid peroxidation, inflammatory signaling, and apoptosis-related pathways. | (7) |
| Enterotoxicity | EBCN | EBCN [4-cyano-4′-ethoxybiphenyl]. | Male ICR mice | 0, 0.2, 2, 20, and 200 µg/kg/day | 35 and 70 days | Environmentally relevant oral exposure model. The 0.2 μg/kg/day dose approximated the dietary exposure dose of LCMs in Chinese adults, and the 2 μg/kg/day dose was close to the estimated daily intake for children based on indoor dust ingestion. | M: Gut microbiota dysbiosis, reduced gut microbial diversity, altered fecal metabolite profiles, decreased anti-inflammatory metabolite L-carnosine, and gut-testis-axis disruption. | (8) |
| Enterotoxicity | Eight-LCM mixture | The mixture contained EtCBN, EBCN, PrCBN, PBIPHCN, PrTFPBC, PrTFMPBC, DFPrPrCB, and FPPrBC. The reported weighting ratios were 24%, 2%, 1%, 15%, 40%, 14%, 4%, and 1%, respectively, based on the proportions of the reported mean concentrations of the individual monomers. The percentages are reproduced as reported in the original study and sum to 101%; no renormalization was performed. | Small-intestinal organoids derived from intestinal crypts of 4-week-old male C57BL/6J mice | 0.3 and 3 μg/mL | 7 consecutive days; exposure medium renewed every 2 days | The minimum concentration of 0.3 μg/mL was selected with reference to reported human serum LCM concentrations of 0.00778–0.276 μg/mL; the higher concentration was used for mechanistic evaluation. | E: Reduced organoid diameter, perimeter, and budding rate; decreased viable-cell proportion; M: Altered expression of stem-cell markers Lgr5, OLFM4, and SMOC2 and differentiation markers Muc2, Villin, and E-cad; impaired intestinal stem-cell renewal and differentiation. | (9) |
| Enterotoxicity | EBCN | EBCN [4-cyano-4′-ethoxybiphenyl] | Small-intestinal organoids derived from intestinal crypts of 4-week-old male C57BL/6J mice | 0.3, 3, and 10 μg/mL | 7 consecutive days; exposure medium renewed every 2 days | The minimum concentration of 0.3 μg/mL was selected with reference to reported human serum LCM concentrations; 3 and 10 μg/mL were used to characterize concentration-related and mechanistic effects. | E: Reduced organoid growth, budding, branch length, and cell viability; increased FITC-dextran permeability; M: Reduced ZO1 and claudin expression; impaired fatty-acid uptake; disrupted intestinal stem-cell homeostasis. H: Complement C3 was proposed as a candidate molecular initiating event within the AOP framework; these findings do not by themselves establish systemic immunosuppression. | (9) |
| Respiratory toxicity | Fourteen selected LCMs | Fourteen LCMs selected from thirty-one LCMs detected in VACF dust based on detection frequency >20%. The toxicologically active compounds reported in the main text included DPrB, 3cH2B, 2OdF3B, MeP3bcH, 2O3cHdFB, 2O2cHdFB, TePT, 2F3T, tFPO-CF2-dF3B, and 2O3cHdFP. | BEAS-2B cells | Individual LCMs: 0.34–34 μg/mL; reconstructed LCM mixture: 0.1–10 units | 24 h | Exposure-informed *in vitro* lung epithelial cell model. Thirty-one LCMs were detected in VACF dust at ΣLCM concentrations of 43.7–448 ng/g. The exposure doses were derived from an EYI model based on VACF dust concentrations and adult dust-ingestion parameters; the mean EYI for the fourteen LCMs corresponded to 3.4 μg/mL for each compound, and one mixture unit corresponded to 4.75 μg. | E/M: Oxidative stress and reduced BEAS-2B cell viability; DPrB, 3cH2B, 2OdF3B, and MeP3bcH caused dose-dependent cytotoxicity, while tFPO-CF2-dF3B, 2O3cHdFP, 2O2cHdFB, and TePT significantly increased ROS generation. | (10) |
| Cutaneous toxicity | Five-LCM mixture | The mixture contained 2O3cHdFB [4-ethoxy-2,3-difluoro-4′-(trans-4-propylcyclohexyl)biphenyl; CAS: 189750-98-9], 33%; tFPO-CF2-dF3B [4-[difluoro(3,4,5-trifluorophenoxy)methyl]-3,5-difluoro-4′-propyl-1,1′-biphenyl; CAS: 303186-20-1], 26%; 2O3cHdFP [1-ethoxy-2,3-difluoro-4-(trans-4-propylcyclohexyl)benzene; CAS: 174350-05-1], 23%; DMPMB [2,3-difluoro-4-[(trans-4-propylcyclohexyl)methoxy]anisole; CAS: 1373116-00-7], 9%; and 2O2cHdFB [4-ethoxy-4′-(4-ethylcyclohexyl)-2,3-difluoro-1,1′-biphenyl; CAS: 323178-01-4], 9%. | HaCaT human keratinocytes | Cytotoxicity screening: 0.5, 10, 20, and 40 μg/mL; subsequent mechanistic analyses mainly used 0.5 and 10 μg/mL | 1–7 days | Concentrations were selected with reference to estimated daily human LCM exposure. However, culture-medium concentrations are not directly equivalent to real-world dermal exposure doses. | E: Time- and concentration-dependent reduction in cell viability; increased ROS generation and DNA damage; reduced basal respiration, ATP production, maximal respiration, and spare respiratory capacity; M: Downregulation of PLOD2 and DDIT4; TNF-pathway activation; and disruption of TCA-cycle, glutathione, and purine metabolism. | (11) |
| Cutaneous toxicity | Five-LCM mixture | Same mixture composition and weighting ratios as above. | Male BALB/c mice, 6–8 weeks old, n = 8/group; topical dorsal-skin exposure in Vaseline | 0, 0.5, and 10 μg/g mixture in Vaseline | 7 days | Repeated topical exposure model used to evaluate direct cutaneous toxicity. Direct equivalence to environmental dermal exposure levels was not established. | M: Increased cutaneous IL-6 and TNF-α levels. E: Skin histopathological abnormalities, including disorganization of skin appendages and adipose tissue. | (11) |
| Endocrine toxicity | Forty-four LCMs | Forty-four LCMs were investigated for endocrine-disruption potential; thirty-eight LCMs were used for inverse virtual screening, and LCM 7, LCM 29, and LCM 41 were further tested in reporter gene assays. LCM 41 was identified as 4′-(octyloxy)-4-biphenylcarbonitrile (CAS: 52364-73-5). | Inverse virtual screening; PPARγ reporter gene assay in HEK 293H cells | Reporter assay: 0.78–100 μM | 16 h exposure for reporter gene assay; antagonist assay included 30 min LCM pre-treatment before rosiglitazone co-exposure | Screening-based endocrine-disruption assessment; tested concentrations were used to characterize receptor-level activity and should be interpreted as *in vitro* screening concentrations rather than direct environmental exposure levels. | M: Potential PPARγ antagonism; LCM 7, LCM 29, and LCM 41 antagonized rosiglitazone-induced PPARγ activation, with LCM 41 showing the strongest PPARγ antagonistic activity. | (7) |
| Endocrine toxicity | Six FLCMs | Same six FLCMs as listed above. | Danio rerio larvae | 2O3cHdFP: 3–300 ng/L; tFPO-CF2-dF3B: 1–100 ng/L; 2O3cHdFB: 0.45–45 ng/L; 2OdFP3bcH: 0.6–60 ng/L; TPrCB: 0.5–50 μg/L; BDPrB: 0.1–10 μg/L | 2–144 hpf | Exposure concentrations were selected based on environmentally relevant concentrations and preliminary toxicity data. | M: Disturbance of thyroid hormone levels, altered HPT-axis-related enzymes and proteins, including TPO, TG, T3, T4, and DIO1/2/3, and disruption of thyroid hormone signaling. | (1) |
| Endocrine toxicity | Six FLCMs | Same six FLCMs as listed above. | GH3 cells | RIC20 values: 2O3cHdFP, 35.1 μM; tFPO-CF2-dF3B, 18.9 μM; 2O3cHdFB, 62.5 μM; 2OdFP3bcH, 32.6 μM; TPrCB, 29.6 μM; BDPrB, 61.2 μM | 24 h | GH3 T-screen assay was used to evaluate thyroid hormone-disrupting activity; cells were exposed to FLCMs at RIC20 concentrations in the absence or presence of T3. | E: Reduced GH3 cell viability. M: Thyroid hormone-disrupting activity; 2O3cHdFB, 2OdFP3bcH, and TPrCB showed T3-antagonistic effects. | (1) |
| Endocrine toxicity | Ten high-priority LCMs and three fluorination-based mixtures | Ten high-priority LCMs, including five nonfluorinated LCMs: LCM-2nf [(trans,trans)-4-propyl-4′-vinyl-1,1′-bi(cyclohexane); CAS: 116020-44-1], LCM-3nf [(trans,trans)-4-methoxy-4′-propyl-1,1′-bi(cyclohexane); CAS: 97398-80-6], LCM-6nf [4-methyl-4′-pentyl-1,1′-biphenyl; CAS: 64835-63-8], LCM-10nf [(trans,trans)-4-(p-tolyl)-4′-vinyl-1,1′-bi(cyclohexane); CAS: 155041-85-3], and LCM-13nf [1-methyl-4-[(trans,trans)-4′-propyl[1,1′-bicyclohexyl]-4-yl]benzene; CAS: 84656-75-7]; and five fluorinated LCMs: LCM-5f [1-ethoxy-2,3-difluoro-4-(trans-4-propylcyclohexyl)benzene; CAS: 174350-05-1], LCM-12f [4-(difluoro(3,4,5-trifluorophenoxy)methyl)-3,5-difluoro-4′-propyl-1,1′-biphenyl; CAS: 303186-20-1], LCM-17f [4-ethoxy-4′-(4-ethylcyclohexyl)-2,3-difluoro-1,1′-biphenyl; CAS: 323178-01-4], LCM-18f [(trans,trans)-4-(4-ethoxy-2,3-difluorophenyl)-4′-propyl-1,1′-bi(cyclohexane); CAS: 123560-48-5], and LCM-21f [4-ethoxy-2,3-difluoro-4′-(trans-4-propylcyclohexyl)-1,1′-biphenyl; CAS: 189750-98-9]. Three fluorination-based mixtures were tested: LCM-ALL mixture containing all ten LCMs, LCM-NF mixture containing the five nonfluorinated LCMs, and LCM-F mixture containing the five fluorinated LCMs. | HEK-293T/17, Ishikawa, and HepG2 cells for luciferase reporter gene assays; 3T3-L1 murine preadipocytes and human bone marrow-derived mesenchymal stem cells (hMSCs) for adipogenesis assays | Individual LCMs: 0.001–10 μM, compound-dependent; LCM-NF and LCM-F mixtures: 0.041–41 μM; LCM-ALL mixture: 0.082–82 μM | Reporter gene assays: 18–24 h; 3T3-L1 adipogenesis assay: 8 days; hMSC adipogenesis assay: 18 days | Exposure-relevant *in vitro* screening model. Concentrations were selected to reflect human exposure scenarios, including serum levels reported in occupationally and nonoccupationally exposed populations and higher tissue concentrations expected from preferential tissue accumulation. | M: Altered nuclear receptor transcriptional activity, including effects on PPARγ, TRβ, RXRα, PR-B, GR, ERα, AR, and AhR; PR-B antagonism by six individual LCMs and all three mixtures; E: Increased triglyceride accumulation and adipogenesis in 3T3-L1 cells and hMSCs; H: Concentration-addition modeling underestimated adipogenic mixture potency, suggesting potential synergistic interactions. | (12) |
| Metabolic toxicity / metabolic reprogramming | Six FLCMs and broader LCM candidates | Six experimental LCMsF: 2teFT [4''-ethyl-2',3,4,5-tetrafluoro-1,1':4',1''-terphenyl; CAS: 326894-55-7]; tFPO-CF2-Tf3t [4''-propyl-4-[difluoro(3,4,5-trifluorophenoxy)methyl]-2',3,5-trifluoro-1,1':4',1''-terphenyl; CAS: 303186-36-9]; 2OdF3B [1-ethoxy-2,3-difluoro-4-(4-propylphenyl)benzene; CAS: 157248-24-3]; 2O3cHdFP [1-ethoxy-2,3-difluoro-4-(4-propylcyclohexyl)benzene; CAS: 174350-05-1]; 5bcHdFB [3,4-difluoro-4'-[4'-pentyl-1,1'-bi(cyclohexyl)-4-yl]biphenyl; CAS: 136609-96-6]; and 3bcHdFB [3,4-difluoro-4'-[4'-propyl-1,1'-bi(cyclohexyl)-4-yl]biphenyl; CAS: 119990-81-7]. | Zebrafish transcriptomics; LCMsT-MTP deep learning model; molecular dynamics validation | 10 μg/L for RNA-seq; preliminary toxicity testing used 0.01, 0.05, 0.20, 0.80, 3.20, 6.40, and 12.8 mg/L | NR for RNA-seq exposure duration; preliminary toxicity testing used 96-h acute exposure | Transcriptomics-informed computational screening model used to prioritize LCMs with potential metabolic toxicity; predictions should be interpreted as screening-level evidence requiring further experimental validation. | M: Predominant metabolic transcriptomic disturbance, 28 KEGG pathway abnormalities, 498 differentially expressed genes. H: Prediction of 21 metabolic toxicity endpoints, and construction of a metabolic toxicity priority list for LCMs. | (13) |
| Metabolic toxicity / metabolic reprogramming | EDPrB, DTMDPB, TPrCB, EDPB, and EDFPBB | Five representative FLCMs: EDPrB [1-ethoxy-2,3-difluoro-4-(4-propylcyclohexyl)benzene; CAS: 174350-05-1]; DTMDPB [2-[difluoro-(3,4,5-trifluorophenoxy)methyl]-1,3-difluoro-5-(4-propylphenyl)benzene; CAS: 303186-20-1]; TPrCB [1,2,3-trifluoro-5-[4-(4-propylcyclohexyl)cyclohexyl]benzene; CAS: 131819-23-3]; EDPB [1-ethoxy-2,3-difluoro-4-[4-(4-propylcyclohexyl)phenyl]benzene; CAS: 189750-98-9]; and EDFPBB [1-ethoxy-2,3-difluoro-4-[4-(4-propylcyclohexyl)cyclohexyl]benzene; CAS: 123560-48-5]. | Zebrafish embryos and larvae | 0.05, 0.5, 5, and 50 μg/L | 6–96 hpf | Early-life zebrafish exposure model used to evaluate sublethal mitochondrial and metabolic disruption; exposure concentrations were analytically verified in exposure solutions. | E: Reduced mitochondrial respiration, ATP-linked oxygen consumption, NAD⁺/NADH ratio, and mitochondrial membrane potential; M: AMPK-associated metabolic reprogramming involving TCA-cycle compensation and amino acid-driven anaplerosis. | (14) |
| Reproductive toxicity | EBCN | EBCN [4-cyano-4′-ethoxybiphenyl]. | Male ICR mice | 0, 0.2, 2, 20, and 200 μg/kg/day | 35 and 70 days | Environmentally relevant oral exposure model designed to assess short-term and long-term reproductive effects of EBCN. | E: Testicular dysfunction, reduced sperm count, sperm morphological abnormalities, decreased Leydig cells and spermatogenic cells, M: Disrupted testosterone/LH/FSH levels, increased serum inflammatory cytokines, and activation of necroptosis-related pathways. | (8) |
| Toxicokinetics / placental transport | Twenty-one target LCMs | Cocktail of twenty-one target LCMs used in the rat uterine perfusion model. Fourteen LCMs detected in human maternal or cord serum included PrEPCC, EDFPrCB, BCBP, PBIPHCN, CFPEB, BDFPrCB, BEEB, EPrCPB, CFPPB, DFPPrBC, DFMPEBC, ETeFT, FPrCB, and DFPrB. | Pregnant rat uterine perfusion model | 0, 10, 1000, and 5000 ng/mL | 55 min perfusion | Ex vivo placental-transfer model. The low dose of 10 ng/mL was equivalent to the concentration range detected in cord serum samples, allowing comparison with human gestational exposure. | TK: Transplacental transfer and placental accumulation of LCMs; M: In complementary mechanistic analyses, SLC16A10-mediated active transport was supported by transporter expression, molecular docking, ITC binding, placental localization, and siRNA knockdown evidence. | (15) |
| Reproductive toxicity | PBIPHCN and LCM mixtures | PBIPHCN, a representative serum-detected LCM; fluorinated and non-fluorinated LCM mixtures detected in maternal or cord serum samples. | BeWo cells, including non-syncytialized and syncytialized BeWo cells | PBIPHCN: 100, 500, 1000, and 2000 μM for cytotoxicity assays; 5, 50, and 500 μM for transcriptomic analysis; LCM mixtures tested at assay-specific concentrations | 24 h for cytotoxicity and transcriptomic assays; 24 and 48 h for progesterone-release assays | PBIPHCN was selected because of its relatively high detection frequency and bioavailability; BeWo placental-cell assays were used to evaluate the relevance of serum-detected LCMs to placental toxicity. | E: Cytotoxicity in placental cells, inhibited trophoblast syncytialization, membrane disruption, decreased hCG release, and reduced progesterone release. M: Reduced hCGB and OVOL1 expression and altered placental-development pathways. | (15) |
| Developmental toxicity | Six FLCMs | Same six FLCMs as listed above. | Danio rerio larvae | 2O3cHdFP: 3–300 ng/L; tFPO-CF2-dF3B: 1–100 ng/L; 2O3cHdFB: 0.45–45 ng/L; 2OdFP3bcH: 0.6–60 ng/L; TPrCB: 0.5–50 μg/L; BDPrB: 0.1–10 μg/L | 2–144 hpf | Developmental exposure model using low, medium, and high concentrations selected based on environmentally relevant concentrations and preliminary toxicity data. | E: Increased larval abnormalities, reduced survival, reduced eye length, and disrupted retinal development. | (1) |
| Immune-relevant hypothesis generation | LCM candidates screened for immune-relevant hazards | LCM candidates evaluated through a machine-learning-assisted immunotoxicity screening framework; compound-level prioritization was linked to 11β-HSD1/HSD11B1-related endocrine–immune regulation. | Machine-learning prediction; molecular docking; targeted mechanistic validation | NR; computational screening with assay-specific validation | NR; assay-specific validation | Screening framework for prioritizing LCM candidates and potential immune-relevant targets. Computational results are hypothesis-generating and are not directly comparable with environmental exposure measurements. | H: Prioritization of LCM candidates with potential immune-related hazards and nomination of 11β-HSD1/HSD11B1 as a candidate target. Computational and docking findings do not by themselves establish functional modulation of 11β-HSD1, altered glucocorticoid signaling, or immune dysfunction *in vivo.* | (16) |

**Notes to Supplementary Table S2**

1. Grouped entries summarized in Supplementary Table S3 are expanded in Supplementary Table S2, including compound identities, exposure regimens, exposure-context information, and major toxicological endpoints.

2. Where environmental or human exposure-context information is unavailable or not directly comparable with the experimental exposure matrix, this is stated explicitly to avoid over-interpreting high-dose *in vitro* screening assays.

3. For grouped compound entries, the “Expanded compound identity” column provides individual compound names, abbreviations, CAS numbers, compound classes, or mixture composition where available from the original studies or their supplementary information. Abbreviations are retained as reported in the original studies; different abbreviations may refer to the same compound, as illustrated by EDPrB and 2O3cHdFP (CAS No. 174350-05-1).

4. Exposure concentrations and durations are reported according to the original experimental design. For mixture exposures, “unit” or “assay-dependent” indicates that the dose was reconstructed or defined according to the source study.

5. E, experimentally observed effects; M, mechanistic findings or associations; H, hypothesis-generating evidence; TK, toxicokinetic observations. These labels indicate evidentiary roles rather than formal evidence-quality scores. Combined labels indicate findings with more than one evidentiary role. Findings apply to the specified models and exposure conditions. Tissue distribution, receptor activity, and computational predictions should not be interpreted as equivalent evidence of organ toxicity or adverse effects in humans.

**Abbreviations and definitions used in Supplementary Table S2**

| Abbreviation | Definition |
| --- | --- |
| LCMs | liquid crystal monomers |
| FLCMs | fluorinated liquid crystal monomers |
| FBAs | fluorinated biphenyl analogues |
| CBAs | cyanobiphenyl analogues |
| BAs | biphenyl analogues |
| VACF | ventilation and air-conditioning filter |
| DF | detection frequency |
| EYI | estimated yearly intake |
| ΣLCMs | sum concentration of liquid crystal monomers |
| ND | not detected |
| NR | not reported |
| hpf | hours post-fertilization |
| h | hours |
| min | minutes |
| d | days |
| bw | body weight |
| RIC20 | concentration causing a 20% reduction in the indicated cell-response readout |
| BEAS-2B | human bronchial epithelial cell line |
| GH3 | rat pituitary tumor cell line |
| BEND3 | mouse brain endothelial cell line |
| BV2 | murine microglial cell line |
| HT22 | mouse hippocampal neuronal cell line |
| RLM | rat liver microsomes |
| HK-2 | human kidney-2 proximal tubular epithelial cell line |
| HEK-293T/17 | human embryonic kidney 293T/17 cell line |
| HEK 293H | human embryonic kidney 293H cell line |
| Ishikawa | human endometrial adenocarcinoma cell line |
| HepG2 | human hepatocellular carcinoma cell line |
| 3T3-L1 | murine preadipocyte cell line |
| hMSCs | human bone marrow-derived mesenchymal stem cells |
| BeWo | human placental choriocarcinoma/trophoblast-like cell line |
| LCM-ALL mixture | mixture containing all ten tested LCMs |
| LCM-NF mixture | mixture containing the five nonfluorinated LCMs |
| LCM-F mixture | mixture containing the five fluorinated LCMs |
| PPARγ | peroxisome proliferator-activated receptor gamma |
| PPARα | peroxisome proliferator-activated receptor alpha |
| PR-B | progesterone receptor B |
| TRβ | thyroid receptor beta |
| RXRα | retinoid X receptor alpha |
| GR | glucocorticoid receptor |
| ERα | estrogen receptor alpha |
| AR | androgen receptor |
| AhR | aryl hydrocarbon receptor |
| HPT axis | hypothalamic-pituitary-thyroid axis |
| TPO | thyroid peroxidase |
| TG | thyroglobulin |
| T3 | triiodothyronine |
| T4 | thyroxine |
| DIO1/2/3 | iodothyronine deiodinase types 1, 2, and 3 |
| AST | aspartate aminotransferase |
| ALT | alanine aminotransferase |
| ER stress | endoplasmic reticulum stress |
| PERK | protein kinase RNA-like endoplasmic reticulum kinase |
| eIF2α | eukaryotic translation initiation factor 2 alpha |
| P53 | tumor protein p53 |
| Bax/Bcl2 | Bcl-2-associated X protein / B-cell lymphoma 2 |
| LDH | lactate dehydrogenase |
| ROS | reactive oxygen species |
| AOP | adverse outcome pathway |
| C3 | complement component 3 |
| LH | luteinizing hormone |
| FSH | follicle-stimulating hormone |
| SLC16A10 | solute carrier family 16 member 10 / aromatic amino acid transporter 1 |
| ITC | isothermal titration calorimetry |
| siRNA | small interfering RNA |
| hCGB | human chorionic gonadotropin beta subunit |
| hCG | human chorionic gonadotropin |
| OVOL1 | ovo-like transcriptional repressor 1 |
| AMPK | AMP-activated protein kinase |
| TCA cycle | tricarboxylic acid cycle |
| KEGG | Kyoto Encyclopedia of Genes and Genomes |
| DEGs | differentially expressed genes |
| LCMsT-MTP | liquid crystal monomer metabolic toxicity prediction model |
| MD | molecular dynamics |
| OCR | oxygen consumption rate |
| MMP | mitochondrial membrane potential |
| NAD⁺/NADH | oxidized/reduced nicotinamide adenine dinucleotide ratio |
| ML | machine learning |
| MIE | molecular initiating event |
| 11β-HSD1 | 11β-hydroxysteroid dehydrogenase 1 |
| HSD11B1 | hydroxysteroid 11-beta dehydrogenase 1 gene |
| DFECB | 3,4-difluoro-4′-(trans-4-ethylcyclohexyl)biphenyl |
| FAO | fatty acid oxidation |
| BUN | blood urea nitrogen |
| HaCaT | human immortalized keratinocyte cell line |
| FITC | fluorescein isothiocyanate |
| ZO1 | zonula occludens 1 |
| E-cad | E-cadherin |
| Muc2 | mucin 2 |
| Lgr5 | leucine-rich repeat-containing G protein-coupled receptor 5 |
| OLFM4 | olfactomedin 4 |
| SMOC2 | SPARC-related modular calcium-binding protein 2 |
| CPT1/2 | carnitine palmitoyltransferase 1/2 |
| ACSL1 | acyl-CoA synthetase long-chain family member 1 |
| GW7647 | PPARα agonist used in the rescue experiment |
| PLOD2 | procollagen-lysine,2-oxoglutarate 5-dioxygenase 2 |
| DDIT4 | DNA damage-inducible transcript 4 |

Supplementary Table S3. Summary of Experimental Toxicological and Toxicokinetic Evidence for LCMs

| Evidence domain | Compounds | Experimental model(s) | Exposure concentration | Exposure duration | Main findings and evidentiary role | References |
| --- | --- | --- | --- | --- | --- | --- |
| Neurotoxicity | Six FLCMs | ‌Danio rerio larvae | 0.45 ng/L–50 μg/L | 2–144 hpf | E: Reduced cell density in retinal layers. | (1) |
| Neurotoxicity | EtCBN | Male C57BL/6J mice | 0, 20, 200 μg/kg | 56 days | E: Cognitive impairment. | (2) |
| Neurotoxicity | EtCBN | BEND3 cells | 0.3, 3, 30 μg/mL | 24, 48 h | E: Brain endothelial-cell cytotoxicity. |  |
| Neurotoxicity | EtCBN | BV2 cells | 0.3, 3, 30 μg/mL | 24, 48 h | E: Microglial-cell cytotoxicity. |  |
| Neurotoxicity | EtCBN | HT22 cells | 0.3, 3, 30 μg/mL | 24, 48 h | E: Hippocampal neuronal-cell cytotoxicity. |  |
| Toxicokinetics | Four target LCMs | Female BALB/c mice | 10 mg/kg bw | 0.5, 1, 3, 6, 24, 72, 168, 336 h | TK: Brain distribution and accumulation; not a direct measure of neurotoxicity. | (3) |
| Toxicokinetics | Thirty-nine target LCMs | Danio rerio | 10 μg/L (mixture) | 19 days | TK: Tissue-specific bioaccumulation, including brain accumulation. | (4) |
| Hepatotoxicity | EDPrB | Adult zebrafish | 0, 1, 10, 100 μg/L | 21 days | E/M: Disruption of hepatic lipid and glucose metabolism. | (5) |
| Toxicokinetics | Four target LCMs | Female BALB/c mice | 10 mg/kg bw | 0.5, 1, 3, 6, 24, 72, 168, 336 h | TK: Preferential liver and adipose distribution; not a direct measure of hepatotoxicity. | (3) |
| Toxicokinetics | Four target LCMs | Rat liver microsomes | 20 μM | 0, 15, 30, 60, 180, and 360 min | TK: Differential metabolic stability in rat liver microsomes. | (3) |
| Nephrotoxicity | DFECB | Female C57BL/6 mice; HK-2 cells; PPARα molecular docking | Mice: 10 mg/kg bw/day;  Cells: 1 nM–500 μM | Mice: 30 days; cells: 24 h | E: Kidney dysfunction. M: Altered PPARα signaling and fatty acid oxidation. | (6) |
| Nephrotoxicity | Forty-four LCMs | HK-2 cells | 20 or 100 μM for selected LCMs | 24 h | M: Alterations in β-oxidation and inflammatory pathways. | (7) |
| Enterotoxicity | EBCN | Male ICR mice | 0, 0.2, 2, 20, 200 μg/kg/day | 35 and 70 days | M: Gut microbiota dysbiosis. | (8) |
| Enterotoxicity | Eight-LCM mixture; EBCN | Mouse small-intestinal organoids | Mixture: 0.3 and 3 μg/mL;  EBCN: 0.3, 3, and 10 μg/mL | 7 days | E: Intestinal barrier injury. M: Altered stem-cell homeostasis. | (9) |
| Respiratory toxicity | Fourteen selected LCMs | BEAS-2B cells | 0.34–34 μg/mL; mixture: 0.1–10 units | 24 h | E: Reduced cell viability. M: Oxidative stress. | (10) |
| Cutaneous toxicity | Five-LCM mixture | HaCaT keratinocytes;  Male BALB/c mice | HaCaT: 0.5, 10, 20, 40 μg/mL;  Mice: 0.5 and 10 μg/g in Vaseline | HaCaT: 1–7 days;  Mice: 7 days | E: Mitochondrial dysfunction and skin histopathological abnormalities. M: Increased cutaneous inflammatory markers. | (11) |
| Endocrine Toxicity | Forty-four LCMs | PPARγ reporter gene assay | 0.78–100 μM | 16 h | M: PPARγ antagonism in a reporter assay. | (7) |
| Endocrine Toxicity | Six FLCMs | ‌Danio rerio larvae | 0.45 ng/L–50 μg/L | 2–144 hpf | M: Altered thyroid hormone levels and HPT-axis-related markers. | (1) |
| Endocrine Toxicity | Six FLCMs | GH3 cells | RIC20: 18.9–62.5 μM | 24 h | M: T3-antagonistic activity in GH3 cells. |  |
| Endocrine Toxicity | Ten high-priority LCMs and three mixtures | HEK-293T/17 cells | Individual LCMs: 0.001–10 μM (compound-dependent); LCM-NF and LCM-F mixtures: 0.041–41 μM; LCM-ALL mixture: 0.082–82 μM | 18–24 h | M: Altered nuclear-receptor reporter activity. | (12) |
| Endocrine Toxicity |  | Ishikawa and HepG2 cells | Individual LCMs: 0.001–10 μM (compound-dependent); LCM-NF and LCM-F mixtures: 0.041–41 μM; LCM-ALL mixture: 0.082–82 μM | 18–24 h | M: Altered nuclear-receptor reporter activity. |  |
| Endocrine Toxicity |  | 3T3-L1 cells and hMSCs | Assay-dependent | 8–18 days | E: Increased triglyceride accumulation and adipogenesis *in vitro.* |  |
| Metabolic toxicity | Six FLCMs | Danio rerio; LCMsT-MTP model | 10 μg/L | NR | M: Metabolic transcriptomic alterations in zebrafish. H: Computational prediction and prioritization of metabolic toxicity. | (13) |
| Metabolic toxicity | Five FLCMs | Danio rerio embryos/larvae | 0.05–50 μg/L | 6–96 hpf | E: Reduced mitochondrial function. M: Metabolic reprogramming. | (14) |
| Reproductive toxicity | EBCN | Male ICR mice | 0, 0.2, 2, 20, and 200 μg/kg/day | 35 and 70 days | E: Testicular dysfunction. | (8) |
| Toxicokinetics / placental transport | Twenty-one target LCMs | Pregnant rat uterine perfusion model | 0, 10, 1000, and 5000 ng/mL | 55 min | TK: Transplacental transfer and placental accumulation. | (15) |
| Reproductive toxicity | PBIPHCN and LCM mixtures | BeWo placental cells | 5–2000 μM | 24–48 h | E: Placental-cell cytotoxicity, impaired trophoblast syncytialization, and reduced progesterone release. |  |
| Developmental toxicity | Six FLCMs | Danio rerio larvae | 0.45 ng/L–50 μg/L | 2–144 hpf | E: Impaired retinal development. | (1) |
| Notes: LCMs: Liquid Crystal Monomers, FLCMs: Fluorinated Liquid Crystal Monomers, NR: Not reported, GH3: Rat pituitary tumor cell line, BEND3: Brain endothelial cell 3, BV2: Murine microglial cell line, HT22: HT22 mouse hippocampal neuronal cells line, RLM: Rat Liver Microsomes, HK-2: Human Kidney-2 cells, BEAS-2B: Human bronchial epithelial cell line, HEK-293T/17: Human Embryonic Kidney 293T/17 cells, HepG2: Human hepatocellular carcinoma cell line, PPARγ: peroxisome proliferator-activated receptor gamma, RNA-seq: RNA sequencing, LCMsT-MTP: liquid crystal monomer metabolic toxicity prediction model, AMPK: AMP-activated protein kinase. PPARα: peroxisome proliferator-activated receptor alpha; HaCaT: human immortalized keratinocyte cell line. Detailed compound identities, exposure regimens, and exposure-context information are provided in Supplementary Table S2.  E, experimentally observed effects; M, mechanistic findings or associations; H, hypothesis-generating evidence; TK, toxicokinetic observations. These labels indicate evidentiary roles rather than formal evidence-quality scores. Combined labels indicate findings with more than one evidentiary role. Findings apply to the specified models and exposure conditions. Tissue distribution, receptor activity, and computational predictions should not be interpreted as equivalent evidence of organ toxicity or adverse effects in humans. | | | | | | |

**References**

1. He S, He J, Ma S, Wei K, Wu F, Xu J, et al. Liquid crystal monomers disrupt photoreceptor patterning of zebrafish larvae via thyroid hormone signaling. Environ Int. (2024) 188:108747. doi: 10.1016/j.envint.2024.108747

2. Duan L, Liang J, Zhang W, Liu Z, Kang H, Huang D, et al. The liquid crystal monomer EtCBN induced neurotoxicity by activating the NOD-like receptor signaling pathway through the Muribaculum-epigallocatechin axis. J Hazard Mater. (2025) 496:139206. doi: 10.1016/j.jhazmat.2025.139206

3. Kong Y, Wen Y, Su G, Peng Y, Cui X. Tissue-specific uptake and distribution of liquid crystal monomers (LCMs) in mice. Environ Int. (2023) 174:107894. doi: 10.1016/j.envint.2023.107894

4. Bao Y, Zhu M, Su G. Tissue-specific accumulation, bioaccumulation, and depuration of liquid crystal monomers (LCMs) in adult zebrafish (Danio rerio). Sci Total Environ. (2023) 859:160267. doi: 10.1016/j.scitotenv.2022.160267

5. Li W, Wang S, Fan H, Wang Z, Liu Y, You H. Insights into hepatotoxicity of fluorinated liquid crystal monomer 1-ethoxy-2,3-difluoro-4-(trans-4-propylcyclohexyl) benzene (EDPrB) in adult zebrafish at environmentally relevant concentrations: Metabolic disorder and stress response. J Hazard Mater. (2025) 486:136858. doi: 10.1016/j.jhazmat.2024.136858

6. Peng L, Xie B, Dai Q, Zhang Y, Zhang J, Kang R, et al. Fluorinated liquid crystal monomer (FLCM) induces kidney dysfunction by disrupting PPARα-mediated fatty acid oxidation: *In vivo*, *in vitro*, and *in silico* assays. Environ Chem Ecotoxicol. (2025) 7:192-200. doi: 10.1016/j.enceco.2024.12.002

7. Zhao H, Li C, Naik MY, Wu J, Cardilla A, Liu M, et al. Liquid Crystal Monomer: A Potential PPARγ Antagonist. Environ Sci Technol. (2023) 57:3758-3771. doi: 10.1021/acs.est.2c08109

8. Wu J, Lv D, Lin W, Mao Y, Xia Y, Feng L, et al. Chronic exposure to liquid crystal monomer EBCN at environmentally relevant concentrations induces testicular dysfunction via the gut-testis axis. J Hazard Mater. (2025) 486:137033. doi: 10.1016/j.jhazmat.2024.137033

9. Zhang W, Duan L, Liang J, Liu Z, Jiang G, Wang Z, et al. Mechanistic Insights into the Effects of Liquid Crystalline Monomers on Intestinal Stem Cell Differentiation Imbalance by Integrating Machine Learning and Adverse Outcome Pathway Framework Based on Organoids. Environ Sci Technol. (2025) 59:18514-18524. doi: 10.1021/acs.est.5c07403

10. Jin Q, Fan Y, Lu Y, Zhan Y, Sun J, Tao D, et al. Liquid crystal monomers in ventilation and air conditioning dust: Indoor characteristics, sources analysis and toxicity assessment. Environ Int. (2023) 180:108212. doi: 10.1016/j.envint.2023.108212

11. Dongye C, Wang S, Chen X, Li C, Zhao Y, Chan TD, et al. Skin toxicity of liquid crystal monomers (LCMs): Mitochondrial dysfunction and metabolic dysregulation revealed by integrated multi-omics analysis. Ecotoxicol Environ Saf. (2026) 312:119953. doi: 10.1016/j.ecoenv.2026.119953

12. Heldman SM, Eccles KM, Kassotis CD. Liquid Crystal Monomers and Their Mixtures Alter Nuclear Receptor Signaling and Promote Adipogenesis *In Vitro*. Endocrinology. (2025) 166:bqaf143. doi: 10.1210/endocr/bqaf143

13. Zhao W, Li J, Hao N, Shi G, Liu J, Deng Z, et al. Integrating RNA sequencing with deep learning-based metabolic toxicity prediction: A new perspective on screening prioritized liquid crystal monomers. J Hazard Mater. (2025) 500:140465. doi: 10.1016/j.jhazmat.2025.140465

14. Wang J, Ma S, Li S, He S, Wei K, Guo X, et al. Mitochondrial dysfunction and AMPK-associated metabolic reprogramming in zebrafish exposed to fluorinated liquid crystal monomers. Ecotoxicol Environ Saf. (2026) 311:119894. doi: 10.1016/j.ecoenv.2026.119894

15. Zhang S, Cheng Z, Zhang T, Ding Y, Zhu H, Wang L, et al. Liquid crystal monomers induce placental development and progesterone release dysregulation through transplacental transportation. Nat Commun. (2025) 16:1204. doi: 10.1038/s41467-025-56552-z

16. Cheng J, Yuan Y, Mu Y, Wu F, Shin HM, Sayes CM, et al. Machine learning-accelerated determination of immunotoxicities of liquid crystal monomers. J Hazard Mater. (2025) 499:140166. doi: 10.1016/j.jhazmat.2025.140166
